# Iron deficiency promotes aortic medial degeneration via destructing cytoskeleton of vascular smooth muscle cells

**DOI:** 10.1002/ctm2.276

**Published:** 2021-01-13

**Authors:** Bowen Li, Zhiwei Wang, Junmou Hong, Yanjia Che, Ruoshi Chen, Zhipeng Hu, Xiaoping Hu, Qi Wu, Junxia Hu, Min Zhang

**Affiliations:** ^1^ Department of Cardiovascular Surgery Renmin Hospital of Wuhan University Wuhan China; ^2^ Cardiovascular Surgery Laboratory Renmin Hospital of Wuhan University Wuhan China; ^3^ Central Laboratory Renmin Hospital of Wuhan University Wuhan China

**Keywords:** aortic medial degeneration, cytoskeleton, iron, metabolism, vascular smooth muscle cells

## Abstract

**Background:**

Aortic dissection (AD) and aortic aneurysm (AA) are critical illnesses with an unclear pathogenetic mechanism that seriously threaten human life. Aortic medial degeneration (AMD) is the main pathological feature of AD and AA. Diseases of iron metabolism can cause a variety of physiological dysfunctions. In this study, we aimed to clarify the state of iron metabolism in patients with AD and AA, and to explore the effect of iron metabolism on AMD.

**Methods:**

A total of 200 patients with AD or AA, and 60 patients with hypertension were included in the study. Blood samples were drawn immediately when patients were admitted to the hospital. Aortic specimens from patients with Stanford type A AD were obtained at the time of surgery. The status of iron metabolism in the circulation and the aortic wall was analyzed. In addition, apolipoprotein E knockout mice were fed chow with a different iron content, and angiotensin II (Ang II) was used to induce AMD. Furthermore, transferrin receptor 1 knockout (TFR1−/−) mice were used to study the effects of iron deficiency (ID) on aortic development, to observe the effects of different iron metabolism status on the formation of AMD in mice, and to explore the cytoskeleton of vascular smooth muscle cells (VSMCs) under different iron metabolism.

**Results:**

Patients with AMD were iron deficient. ID is associated with the development of AMD in hypertensive patients. Iron‐deficient feeding combined with Ang II pumping promoted the formation of AMD and significantly shortened the survival time of mice. ID significantly impaired the cytoskeleton of VSMCs.

**Conclusions:**

: Our results highlighted that ID was associated with the formation of AMD in patients with hypertension. In this study, we identified a novel mechanism behind VSMCs dysfunction that was induced by ID, thereby suggesting iron homeostasis as a future precaution in patients with hypertension based on its important role in the maintenance of VSMC function.

## Graphical abstract

When iron deficient, the expression of IGTB and Cdc42 in vascular smooth muscle cells (VSMCs) is reduced, while the expression of Rac‐1 is increased. In addition, the arrangement of F‐actin is disordered, it gathers toward the cell edge, and the polarity disappears, resulting in destruction of the cytoskeletal structure and dysfunction, breakage of elastic fibers, increase in collagen deposition, and ultimately the formation of aortic medial degeneration (AMD).

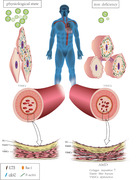

## 1 BACKGROUND

Patients with hypertension have an increased risk of aortic diseases, including aortic aneurysm (AA) and aortic dissection (AD), which are life‐threatening conditions.^1^, ^2^ Aortic medial degeneration (AMD) is the main pathological feature of AA and AD.^3^ Currently, the pathogenesis of AMD is still unclear; therefore, its prevention is restricted. In previous studies, it was indicated that dysfunction of vascular smooth muscle cells (VSMCs) plays a major role in the formation of AMD, while the initiating factor remains to be elucidated.^4^ Abnormalities in trace element metabolism have been shown to cause cell dysfunction and impair the stability of tissue or organ structure.^5^, ^6^ Therefore, maintaining the metabolic homeostasis of trace elements in patients with hypertension is potential precautional target for AMD.

Iron is the most abundant trace element in the human body, and iron metabolism disorder is one of the most common diseases in humans.^7^ Cells must maintain an adequate supply of iron for key physiological and developmental processes, while at the same time restricting the size of a labile iron pool to prevent excessive generation of reactive oxygen species (ROS) from Fenton‐type reactions.^8^, ^9^ In previous studies, it was shown that iron deficiency (ID) plays a pivot role in the development of cardiovascular diseases.^10^ Partly, ID affects the heart by reducing muscle exercise capacity, and limiting the availability of oxygen for oxidative phosphorylation within cardiomyocytes.^11^, ^12^ In the context of chronic heart failure, clinical outcomes are worse in non‐anemic iron‐deficient patients when compared to anemic iron‐replete patients, suggesting that ID may directly affect cardiomyocytes in a manner that is distinct from the effect of anemia.^13^ Few studies have reported the effects of iron metabolism on aortic diseases, however, reports on iron metabolism status vary. In some studies, it was hypothesized that aortic wall erosion may be promoted by high oxidative stress and inflammation because of high iron levels and macrophage infiltration in abdominal AA when compared to no aortic abdominal AA tissue samples.^14^, ^15^, ^16^ In two large sample studies, it was found that levels of circulating iron and TFR were decreased, and that red blood cell‐borne iron retention and transferrin receptor (TFR) levels were increased in abdominal AA tissue.^17^, ^18^ In addition, Marie and colleagues found that iron levels were significantly increased in the serum of thoracic AD patients, while in the aortic tissue, iron levels tended to increase but were not significantly different. Moreover, their data did not support the hypothesis that inflammation is involved in the pathogenesis of thoracic AD.^19^ None of the studies mentioned above explored the underlying mechanism involved in the occurrence of AMD caused by iron metabolism disorders. Instead, studies involved functional studies and focused on whether iron metabolism disorders can lead to the development of aortic diseases. In our preliminary studies, we found that the iron content in the circulation and the pathological aortic medial of patients with AMD was reduced.

Therefore, the present study was designed to test the hypothesis that ID may be related to the pathogenesis of AMD in patients with hypertension. We demonstrated that compared with hypertensive patients, the iron content in both the circulation and aortic tissue of patients with AMD was decreased. ID feeding significantly promoted the formation of AMD in mice. In addition, we revealed that ID induced AMD by destructing the cytoskeleton of VSMCs.

## 2 MATERIALS AND METHODS

### 2.1 Study population

All experimental protocols regarding human materials were conducted according to the Declaration of Helsinki, and were approved by the ethical committee of Renmin Hospital of Wuhan University (WDRY2015‐K021) (Wuhan, China). The trial was registered on the Chinese Clinical Trial Registry (ChiCTR1800014384). All subjects were informed of the purpose of the trial and gave oral and written consent.

Two hundred patients with AD or AA, and 60 patients with hypertension were included between June 5, 2017, and September 7, 2018 at Renmin Hospital of the Wuhan University (Wuhan, China). Clinically unstable patients, patients with hereditary or traumatic aortic disease, contraindications to computed tomography angiography, or patients who were unable to provide informed consent were excluded. Baseline characteristics of the AMD patients and hypertension patients are presented in Table 1.

**TABLE 1:** Clinical characteristics of patients

	Aortic diseases group (n = 200)	Control group (n = 60)	*P*‐value
Age (years)	59.28 ± 13.28	52.74 ± 11.89	.0007**
Male/female (n)	160/40	29/31	<.0001***
BMI	28.46 ± 5.026	21.18 ± 2.678	<.0001***
Systolic blood pressure (mmHg)	172.7 ± 2.010	143.2 ± 1.145	<.0001***
Drinking history (n)	168	48	.5558
<100 g/d (n)	45	32	<.0001***
101‐150 g/d (n)	27	10	.5145
>150 g/d (n)	96	6	<.0001***
Smoking history (n)	166	43	.0638
<10c/d (n)	23	8	.4714
10‐20c/d (n)	121	29	.5687
>20c/d (n)	22	6	1.0000
Drug use history (n)	0	0	1.0000
Family history of aortic diseases (n)	0	0	.7525
LVEF (%)	58.04 ± 4.101	57.05 ± 6.358	.1562
Ultrasound‐detected liver lesions (n)	172	7	<.0001***
Ultrasound‐detected kidney lesions (n)	56	15	.7420
Comorbidities (n)			
Hypertensive disease	178	60	.0031**
Diabetes mellitus	18	19	<.0001***
Gastrointestinal disease	17	7	.4516
Neurodegenerative disease	0	0	1.0000
Type of aortic disease (n)	200	0	<.0001***
Type A AD	66	0	
Type B AD	88	0	
AA	46	0	

Values are mean ± SD or n.

Abbreviations: AA, aortic aneurysm; AD, aortic dissection; BMI, body mass index; LVEF, left ventricle ejection fraction.

*p < .05.

** *p* < .01.

*** *p* < .0001 versus control group.

### 2.2 Detection of iron metabolism in blood

Blood samples for analysis of iron metabolism were drawn as soon as patients were admitted to the hospital. Blood was centrifuged within 1 hour of being drawn at 2500 g for 10 minutes at room temperature, and serum was collected and stored at −80℃.Serum levels of iron were quantified by the serum iron assay kit (Nanjing Jiancheng Bioengineering Institute, Nanjing, China). Routine biochemical analyses, including TF and the soluble TFR (sTFR), were determined by conventional assays. Bottom and peak levels of these biochemical molecular analyses were defined as the minimum and maximum value measured during hospitalization. The inter‐assay and intra‐assay coefficient of variation were <7% for all the latter analyses.

### 2.3 Prussian blue assay

Fifteen pathological tissues from the ascending aorta were obtained from patients who underwent ascending aorta replacement, while the same segment of normal tissue was derived from organ donors with hypertension but no signs of aortic diseases (n = 15). According to a previously described study,^15^ tissue samples were fixed with 4% paraformaldehyde/0.1 M phosphate buffered saline for 24 hours. Then, fixed sections were dehydrated, embedded in paraffin, sectioned to 4μm, and dewaxed, and incubated with ∼50 μL of dilute hydrochloric acid/potassium ferrocyanide solution at room temperature for 15‐20 minutes. Then, sections were stained with ∼50 μL nuclear fast red solution for 15‐30 seconds. Finally, sections were stained with hematoxylin and dehydrated with gradient alcohol and xylene.

### 2.4 H&E staining

Aortic tissues embedded in paraffin were deparaffinized as mentioned above and stained with hematoxylin for 10 minutes. After incubation with hydrochloric acid alcohol solution and ammonium hydroxide for 30 seconds, sections were stained with eosin for 3 minutes. An alcohol gradient using an increasing concentration of alcohol was used to dehydrate the sections. Next, sections were treated three times 3 minutes with xylene. Finally, neutral balsam was used for mounting.

### 2.5 Immunohistochemistry assay

Paraffin sections were dewaxed and hydrated, then hydrated with 3% hydrogen peroxide to quench endogenous peroxidase. Subsequently, sections were incubated with 50 μL (1:100 dilution) rabbit/mouse primary antibody overnight at 4℃. Sections were washed and incubated with 50 μL anti‐rabbit/mouse immunoglobulin G antibody for 1 hour at 37℃ and developed with diaminobenzidine. Finally, sections were stained with hematoxylin and dehydrated with gradient alcohol and xylene. Immunohistochemical analysis was conducted as previously described.^20^

### 2.6 Elastic van Gieson assay

Sections were dewaxed and hydrated, stained with Verhoeff's hematoxylin solution at room temperature for 30 minutes, and then differentiated with 2% ferric chloride solution until black fibers and gray background were observed under the microscope. Subsequently, 5% thio sodium sulfate was used to remove iodine for 1 minute, followed by counterstaining with Van Gieson's solution for 5 minutes. Then, sections were dehydrated, xylene transparent, and mounted. Using a light microscope, the black parts indicate elastic fibers, and the red parts indicate collagen.

### 2.7 F‐actin analysis

Aortic tissues embedded in paraffin were deparaffinized as mentioned above and incubated with iFluor488‐phalloidin for 60 minutes at room temperature, then nuclei were stained with DAPI for 10 minutes in the dark, and images were captured.

### 2.8 Western blot analysis

Protein in aortic medial tissues or VSMCs were extracted as previously reported.^4^ Equal amounts of protein (10μg) were resolved by sodium dodecyl sulfate‐polyacrylamide gel electrophoresis, transferred onto polyvinylidene fluoride membranes, blocked with 5% non‐fat milk for 1 hour at room temperature, and incubated with primary antibodies directed against TFR1 (Abcam, 1:1000, ab84036), TF (Proteintech, 1:1000,17435‐1‐AP), ITGB (Proteintech, 1:1000,12594‐1‐AP), Rac‐1 (Abcam, 1:1000, ab33186), Cdc42 (Abcam, 1:1000, ab64533), phosphor myosin light chain (MLC) (Abcam,1:1000, ab2480), and a‐tubulin (Proteintech, 1:5000,11224‐1‐AP) overnight at 4℃. Then, membranes were incubated with a secondary antibody (LI‐COR, 1:10,000) for 1 hour at room temperature, and signals were visualized by incubation with an enhanced chemiluminescence reagent (Odyssey).

### 2.9 Animal protocol and analysis

Male ApoE‐/‐ mice (4 months of age, 20‐22 g) were used to conduct in vivo studies. To induce AD, 1 μg/kg/min angiotensin II (Ang II) subcutaneous pumping was performed for 4 weeks (Ang II) as previously described.^21^ ID was induced in mice using a chow within an iron content of 5 ppm (ID). A low‐iron diet supplemented with Ang II that was subcutaneously pumped into ApoE‐/‐ mice was used to simulate hypertension with ID (ID+ Ang II). Mice in the control group were fed a chow with a normal iron content (control). The relationship between ID and survival time and the AD rate in mice was analyzed. To investigate the role of ID on aortic development, TFR1 gene knockout (KO) mice were constructed. Fetal mice were harvested at different time points, and various pathological tests were performed on the aorta to investigate the changes in aortic structure and the expression of different aortic development markers.

## 3 IRON INTERVENTION AND FUNCTIONAL EXPERIMENTS

Human aortic smooth muscle cells were obtained from ATCC. In brief, cells were cultured in Dulbecco's Modified Eagle's Medium containing 10% fetal bovine serum, 1% mycillin, 2 mM glutamine, 50 μg/mL gentamycin, and 50 μg/mL amphotericin‐B at 37℃ in a 5% CO_2_ atmosphere. Deferoxamine was used to establish an iron deficient medium, and then add ferrous sulfate to achieve final concentrations of iron in the medium of 0 μM, 20 μM, 50 μM, and 100 μM, respectively. Since TFR is mainly present in the liver, 10 μM of TF was added to each medium to facilitate the entry of iron into the cells based on different iron concentrations. Next, the cells were stimulated with 0.1 M Ang II to investigate the function and structure of VSMCs as previously described.^4^

### 3.1 Statistical analysis

Statistical analysis was performed using GraphPad Prism6 software. Concentration‐response curves were analyzed by two‐way analysis of variance with repeated measurement. Depending on the distribution of data, *t*‐test or nonparametric equivalent was performed for comparison between two groups. Analyses of 3 or more groups were performed using one‐way analysis or variance. Data are presented as the mean ± SEM unless otherwise indicated. Associations between concentrations of iron and aortic diseases were assessed by both correlation analyses and group analyses. A forward logistic regression model was employed to investigate whether iron concentration was an independent risk factor for aortic disease. Values of *p < .05, **p < .01, and ***p < .001 were considered statistically significant.

## 4 RESULTS

### 4.1 Basal characteristics

Basal characteristics are presented in **Table** **1**. Patients with AMD had a higher body mass index, and the proportion of patients with a high alcohol consumption (>150 g/d, > three times/w) and with ultrasound‐detected liver lesions in AMD was higher compared to patients with hypertension. Furthermore, the proportion of patients with a history of diabetes was lower compared to that of hypertension.

### 4.2 Patients with AMD were iron deficient without anemia

**FIGURE 1:**
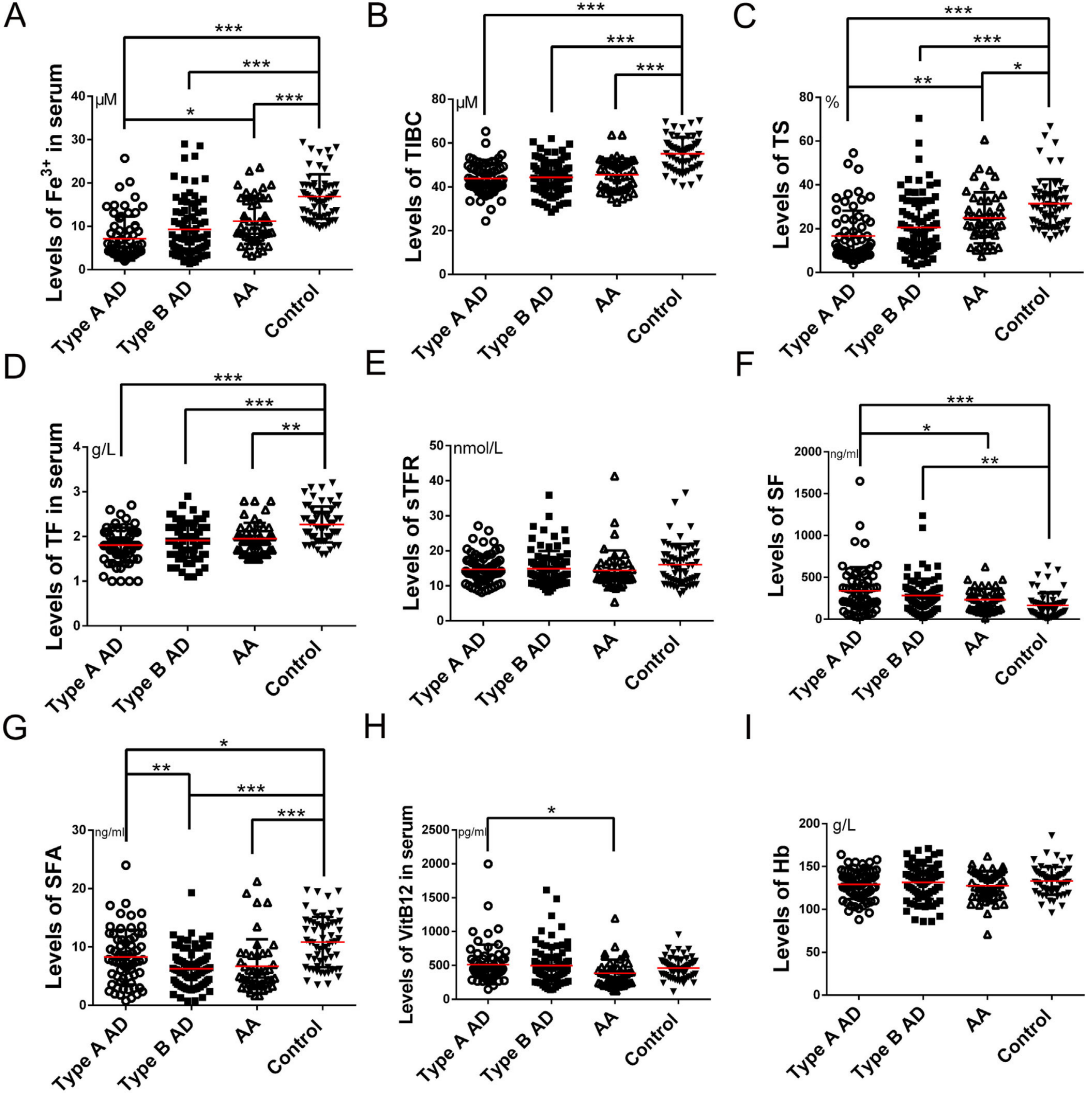
Patients with aortic diseases were iron deficient without anemia. (A) The total iron binding capacity (B), transferrin saturation (C), and transferrin (D) were lower in patients with aortic diseases compared to control patients. E, No significant differences were observed in serum transferrin receptor concentrations between control patients and patients with aortic diseases, while ferritin levels were higher in patients with acute aortic diseases (F). (G‐I) Although serum folate was lower in patients with aortic diseases compared with control patients, differences in vitamin B12 and hemoglobin concentrations were not statistically significant. N _type A AD _= 66, N _type B AD _= 88, N _AA _= 46, and N _control _= 60. Values are the mean ± SEM. *p < .05, **p < .01, ***p < .0001 versus patients with hypertension (control group) by one‐way analysis of variance

From 260 patients, blood samples for iron metabolism analyses were available. The indicators of iron metabolism are presented in Figure 1. The data illustrate a significant decrease in levels of circulating iron (Fe^3+^), total iron binding capacity (TIBC), TF, and transferrin saturation (TS) (Figures 1A‐1D), while the levels of ferritin were significantly increased in the serum of patients with AMD compared to patients with hypertension (Figure 1F). Levels of C‐reactive protein, D‐Dimer, and blood glucose in patients with acute AD were higher than those in patients with AA and hypertension (Figures 1C, 1H, and 1O). However, levels of hemoglobin (Hb) were not significantly different (Figure 1I). Taken together, these data indicate that there was ID in the circulation of patients who presented with AMD without anemia.

### 4.3 ID is an independent risk factor for AMD in hypertensive patients

Correlation analysis showed that the iron concentration negatively correlated with AMD in hypertensive patients (Table 2). Logistic regression analysis was performed, suggesting that ID was an independent risk factor for AMD in hypertensive patients (Table 3). Furthermore, multivariate logistic regression analyses indicated that ID was a risk factor for AD rather than AA in hypertensive patients (Table S1).

**TABLE 2:** Correlation between concentration of iron and aortic disease

Control variable			Fe	Disease
Age and CRP and PT	Fe^3+^	Correlation	1.000	−0.265^a^
D‐Dimer and FER and BMI and Sex and Alcohol and Blood pressure		*P*‐value		0.000***
		Df	0	249

Abbreviations: BMI, body mass index; CRP, C‐reaction protein; FER, Ferritin; PT, prothrombin time; TF, transferrin.

^a^ Iron concentration is negatively correlated with aortic disease.

*** Iron concentration is significantly associated with aortic disease.

**TABLE 3:** Logistic regression analysis of iron concentration and aortic disease

	Β	S.E.	Wald	Df	*P*‐value	OR	95% CI
Age	0.103	0.031	11.147	1	.001*	1.108	1.043‐1.177
PT	0.396	0.155	6.504	1	.011*	1.486	1.096‐2.015
D‐Dimer	0.506	0.146	12.016	1	.001*	1.658	1.246‐2.206
Fe3+	−0.191	0.048	15.965	1	.000***	0.826	0.752‐0.907
BMI	0.529	0.118	19.990	1	.000***	1.698	1.346‐2.141
Sex	−2.383	0.718	11.002	1	.001*	0.092	0.023‐0.377
Blood pressure	0.441	0.128	13.843	1	.001*	1.544	1.198‐2.174
Constant	‐17.379	4.394	15.643	1	.000	0.000	

Abbreviations: BMI, body mass index; PT, prothrombin time.

### 4.4 It is iron deficient in AMD specimens

**FIGURE 2:**
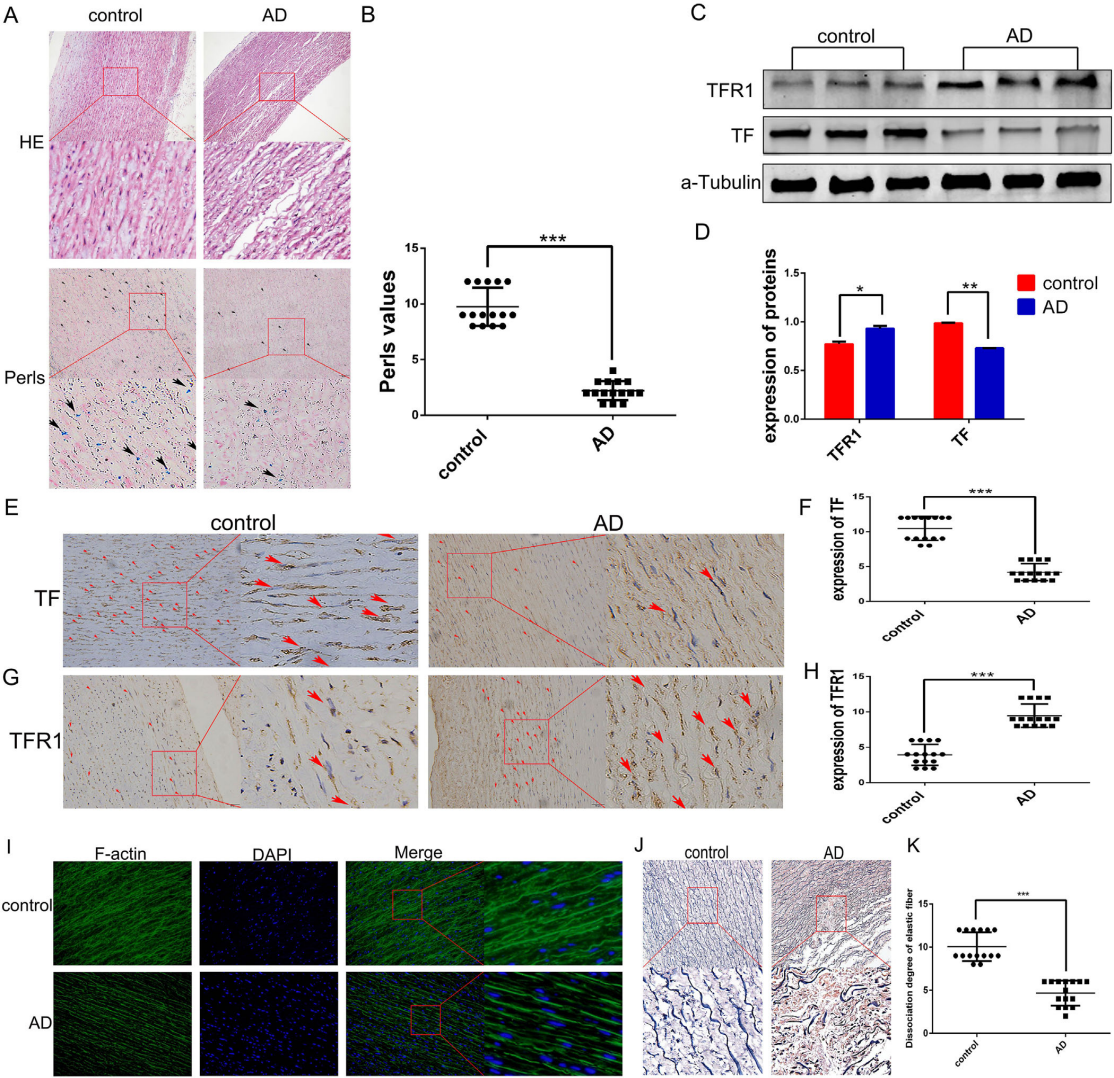
(A and B) There was less iron deposition in aortic medial degeneration specimens (*N* = 15). The expression of transferrin (TF) was lower in pathological aortic tissues, while the trend of TFR1 expression was reversed (C‐H). I, Compared with the control group, the expression of F‐actin in aortic specimens of aortic medial degeneration (AMD) patients was lower, and the distribution was disordered. (J and K) Elastic fibers (black) disintegrated and ruptured, and collagen deposition (red) increased in AMD specimens. Values are the mean ± SEM. *p < .05, **p < .01, ***p < .0001 versus tissues from aortic donor with hypertension (control group) by *t*‐test analysis of variance

Next, we investigated the state of iron metabolism in AMD specimens and the normal aorta, respectively. Prussian blue staining showed that the iron deposition in the aortic tunica media of pathological aortic tissues was significantly less compared to that in normal aortic tissue (Figure 2A). In addition, we determined the expression of iron metabolism‐related proteins by western blot analysis and immunohistochemistry (IHC). The results showed that TF expression was decreased in AMD specimens, while levels of TFR 1 (TFR1) were increased (Figures 2C‐2H). Moreover, we found that the TFR1 gene also increased in the aortic specimens. However, since TF is mainly synthesized in the liver, no mRNA expression of TF in aortic specimens was detected by polymerase chain reaction (Figure S2A). In addition, among the 15 AMD specimens tested, a large amount of iron accumulation was detected in the aortic tissue of one specimen. This iron accumulation region almost completely coincided with the positive region indicated by CD68 staining. We believe that the iron accumulation found was due to local macrophage aggregation (Figure S2B). Our IHC results indicated that no significant differences were observed in the expression of CD68 between control and AD aorta samples (Figure S2C‐D). The Verhoeff's van Gieson (EVG) staining showed that in the AD specimens, the elastic fibers collapsed (black), and collagen deposition (red) increased (Figures 2J and 2K). Furthermore, we observed a significant reduction in the amount of F‐actin (Figure 2I) and phosphorylated MLC (p‐MLC) (Figure. S2E) in AMD tissue compared to normal aortic specimens. Taken together, these observations suggest that It is iron‐deficient in AMD specimens, accompanied by disturbances in matrix components of the middle layer of aortic wall and dysfunction of VSMCs.

### 4.5 Low iron feeding and Ang II induction have a synergistic effect on the formation of AMD in mice

**FIGURE 3:**
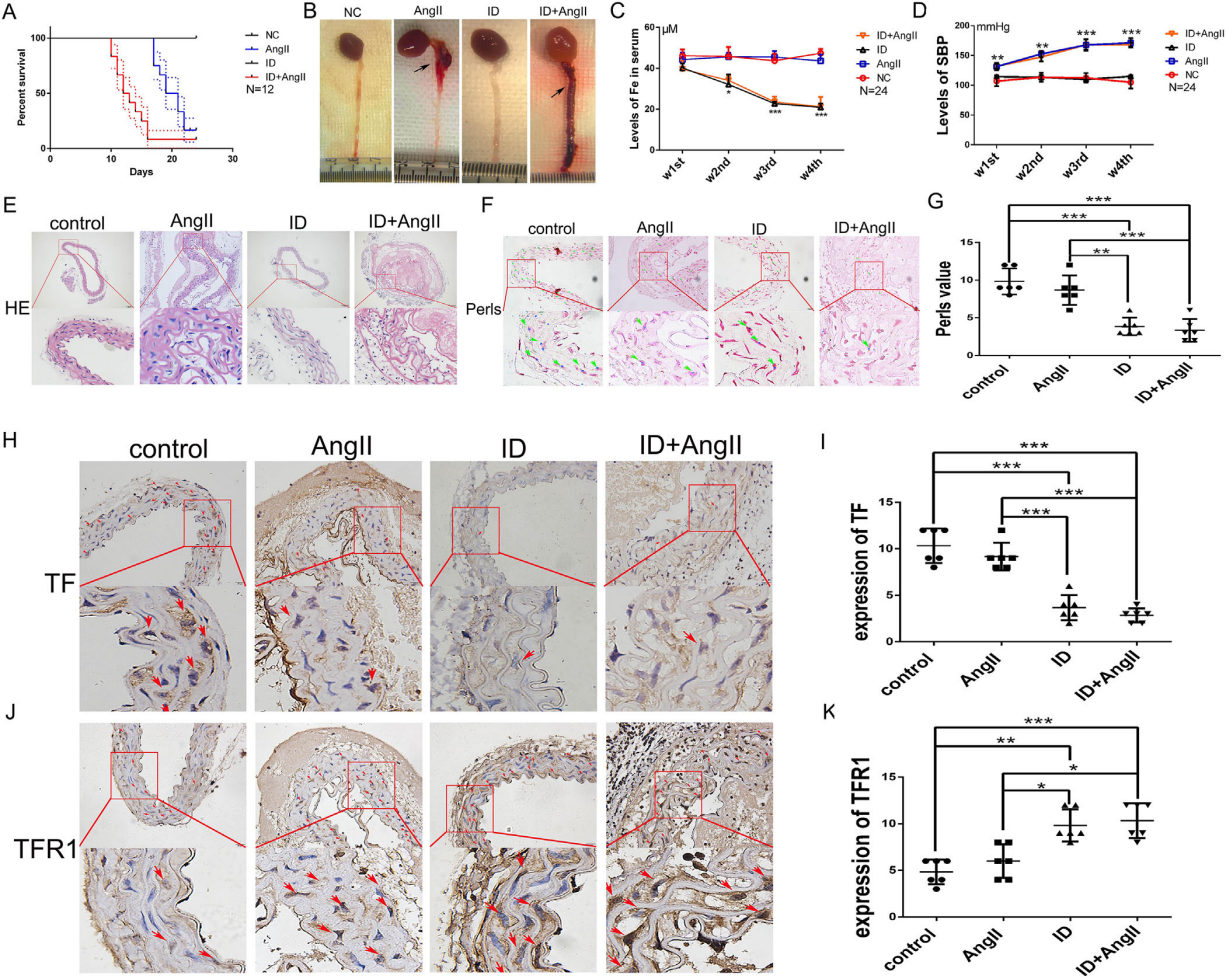
Iron deficiency increased the incidence and severity of aortic medial degeneration in mice. (A) A low iron diet combined with Ang II administration significantly reduced the survival time in mice compared with subcutaneous administration of Ang II alone. The low iron diet alone did not affect survival in mice, and (B) the extent of tearing of the AD was increased in mice with an iron deficiency combined with Ang II induction (*N* = 6). In the second week of low‐iron feeding, mice were found to have an iron deficiency, and the iron deficiency increased with time, but did not affect the systolic blood pressure (C and D, *N* = 6 × 4 weeks). The aortic tissue of AMD mice was disordered (E) and iron deposition was reduced in aortic tissues of low‐iron‐fed mice. Iron deposition in the aorta of mice with Ang II‐induced AMD was reduced, however, no significant differences were observed compared with the control group. (F and G, *N* = 6). H‐K, The expression of transferrin (TF) in the aorta of iron‐deficient mice was decreased, while the expression of TFR1 was increased (*N* = 6). Values are the mean ± SEM. *p < .05, **p < .01, ***p < .0001 versus aortic tissues from mice with different interventions by one‐way analysis or versus serum from different interventions at different time points by two‐way analysis of variance

**FIGURE 4:**
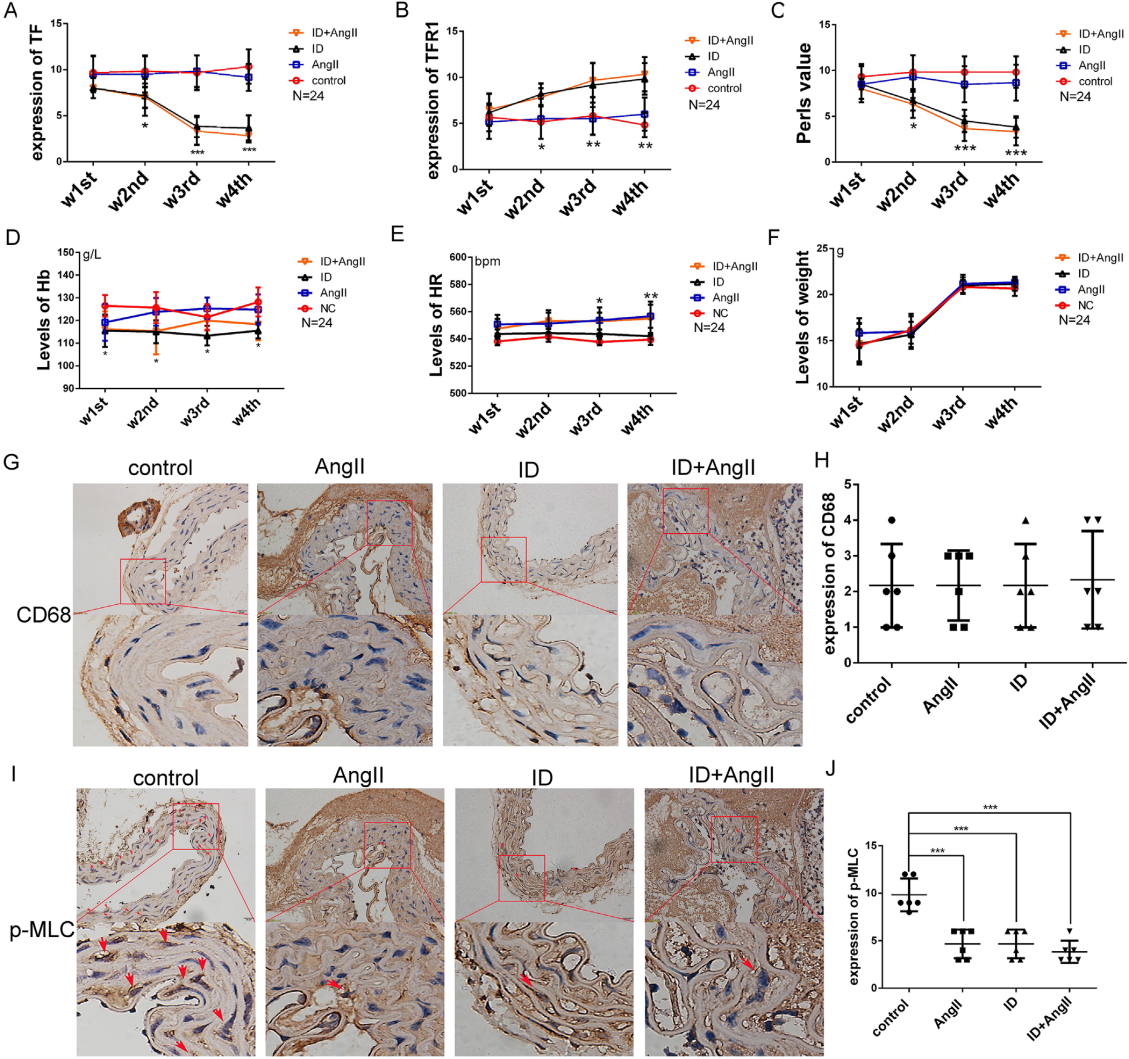
Iron deficiency aggravated Ang II‐induced aortic damage. From the second week of low‐iron feeding, the expression of transferrin (TF) (A) and iron deposition (C) in the aorta of iron‐deficient mice decreased, while the expression of TFR1 (B) gradually increased. Low iron feeding caused a decrease in hemoglobin levels in mice (D), and Ang II administration increased the heart rate in mice (E). Different interventions did not affect the body weight of mice (F). G‐J, No significant differences were observed in the expression of CD68 in the aorta of mice in different intervention groups, while the expression of p‐MLC was significantly decreased in the aorta of Ang II‐infused mice and iron‐deficient mice (*N* = 6). Values are the mean ± SEM. *p < .05, **p < .01, ***p < .0001 versus aortic tissues derived from mice that underwent different interventions by one‐way analysis or versus serum from different intervention at different time points by two‐way analysis of variance

To investigate whether ID was involved in the formation of AMD, apoE‐/‐ mice were fed a low‐iron feed, and AMD was induced by subcutaneous infusion of Ang II. The survival time of mice with low iron combined with Ang II infusion was significantly shorter compared to that of Ang II infusion alone. Furthermore, the AD tears in mice were more extensive, but compared with the control group, ID alone did not affect the survival time of mice (Figures 3A and 3B). The iron content in the circulation of low‐iron‐fed mice decreased significantly from the second week and further decreased over time, but did not affect the blood pressure (Figures 3C and 3D). In addition, low iron‐feeding reduced the iron content in the aortic wall, reduced TF expression, and increased TFR1 expression (Figures 3F‐3K). The changes in iron deposition and protein expression corresponded to the changes in iron content in the circulation (Figures 4A‐4C). Inconsistent with human blood tests, low iron‐fed mice showed a decrease in Hb content, without affecting the heart rate, and body weight in mice (Figures 4D‐4F). These results indicated that ID does not affect the blood pressure of mice, but increased the incidence and severity of AMD.

### 4.6 ID destructed the cytoskeleton of VSMCs in vivo

**FIGURE 5:**
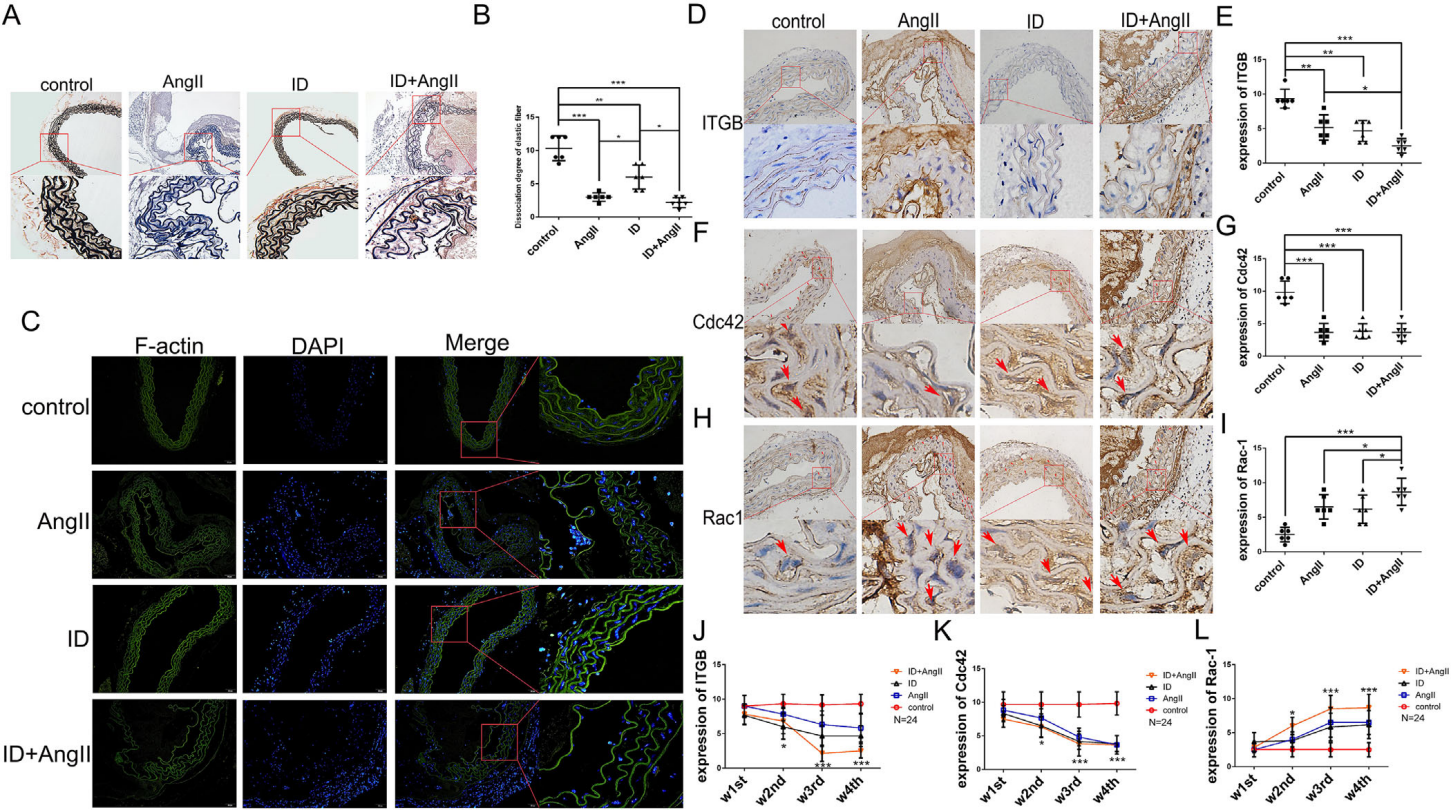
(A and B) In the aortic tissue of aortic medial degeneration mice, the elastic fibers disintegrated and ruptured, and collagen deposition in the aorta of mice that was impaired by iron deficiency combined with Ang II was increased. Both Ang II administration and iron deficiency impaired the expression and distribution of F‐actin in mouse aortic tissue (C). Both Ang II administration and iron deficiency reduced the expression of ITGB and Cdc42 in mouse aortic tissue, but increased the expression of Rac1. In addition, iron deficiency further aggravated Ang II damage to the aorta (D‐I). From the second week of intervention, the expression of ITGB (J) and Cdc42 (K) in the aorta of the intervention group gradually decreased compared with mice in the control group, while the expression of Rac1 showed an opposite trend (L). *N* = 6, values are the mean ± SEM. *p < .05, **p < .01, ***p < .0001 versus aortic tissue from mice that underwent different interventions by one‐way analysis of variance

**FIGURE 6:**
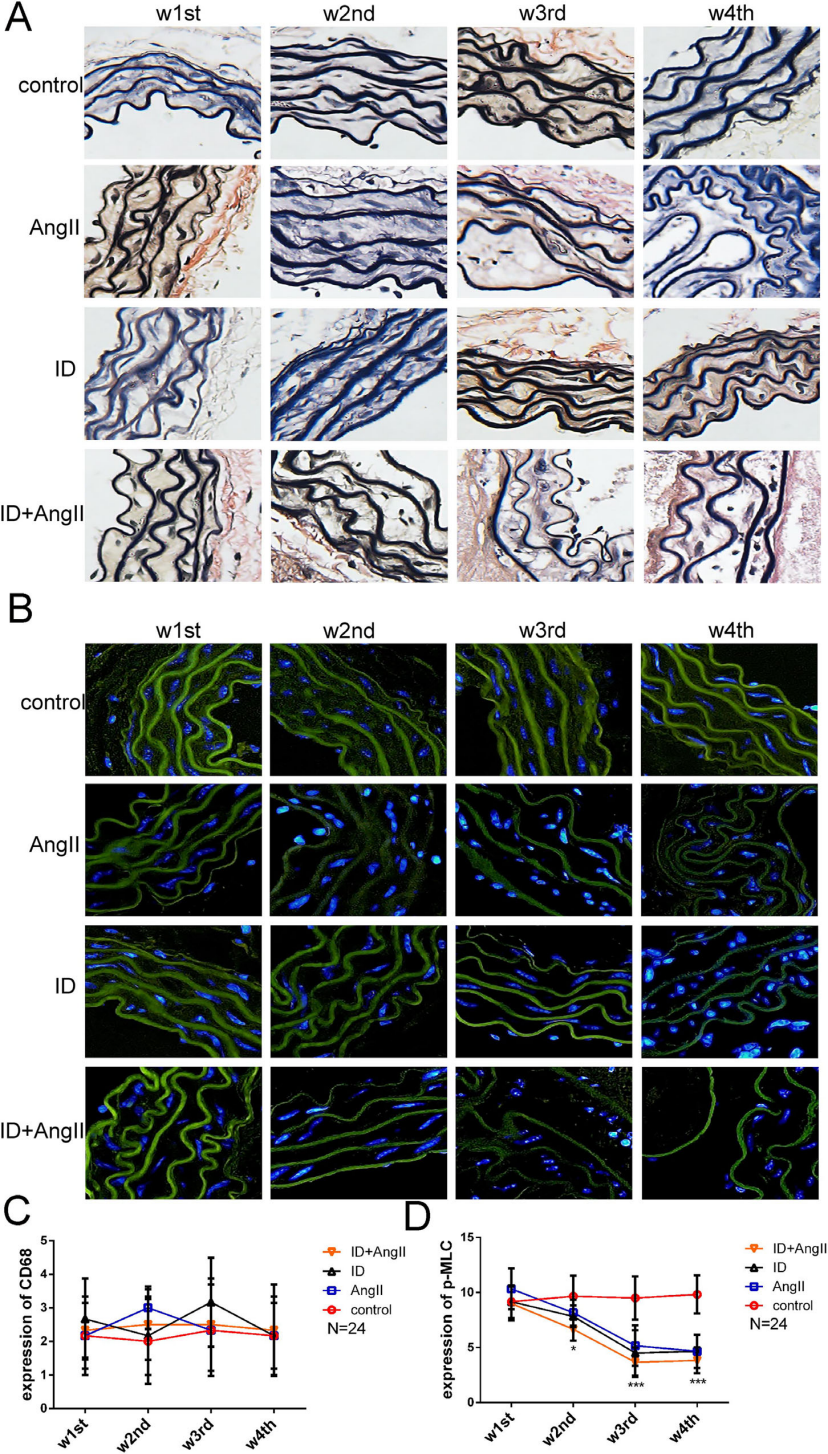
(A) Accompanied by iron deficiency in mice,the continuity of the elastic fibers was destroyed, and the rehearsal was gradually disordered. In addition, the expression and distribution of F‐actin in the aorta abnormally and gradually increased from the second week of iron deficiency intervention (B). The difference in expression of CD68 was not statistically significant (C), while the expression of p‐MLC (D) in the aorta of the intervention group gradually decreased compared with mice in the control group. Values are the mean ± SEM. *p < .05, **p < .01, ***p < .0001 versus aortic tissues from mice that underwent different interventions at different time points by two‐way analysis of variance

Similar to the results shown in human tissue specimens, elastic fibers (black) in the aortic tissue of mice with AMD disintegrated and fractured, the collagen deposition (red) increased, and the F‐actin content and p‐MLC expression were significantly reduced and without a difference in CD68 expression (Figures 4G‐4J, Figures 5A‐3C). These changes gradually deteriorated with the severity of ID (Figures 6A‐6D). F‐actin is one of the main proteins that maintain the cytoskeleton. The degree of adhesion between cells and cells, and between cells and extracellular matrix is an important factor affecting the cytoskeleton. Therefore, in addition to detecting the F‐actin content, we also examined protein expression of the integrin pathway in the aorta. The results suggested that the expression of integrin β3 (ITGB) and Cdc42 was significantly decreased and that Rac1 expression was increased not only in AD mice but also in the aorta of low‐iron‐alone‐fed mice. The above‐mentioned changes also corresponded to the degree of ID (Figures 5D‐5L). The above‐mentioned data suggest that ID can indeed aggravate AMD, and may be caused by regulation of the cytoskeleton and the function of VSMCs.

### 4.7 ID resulting in poor vascular development in TFR1 KO mice

**FIGURE 7:**
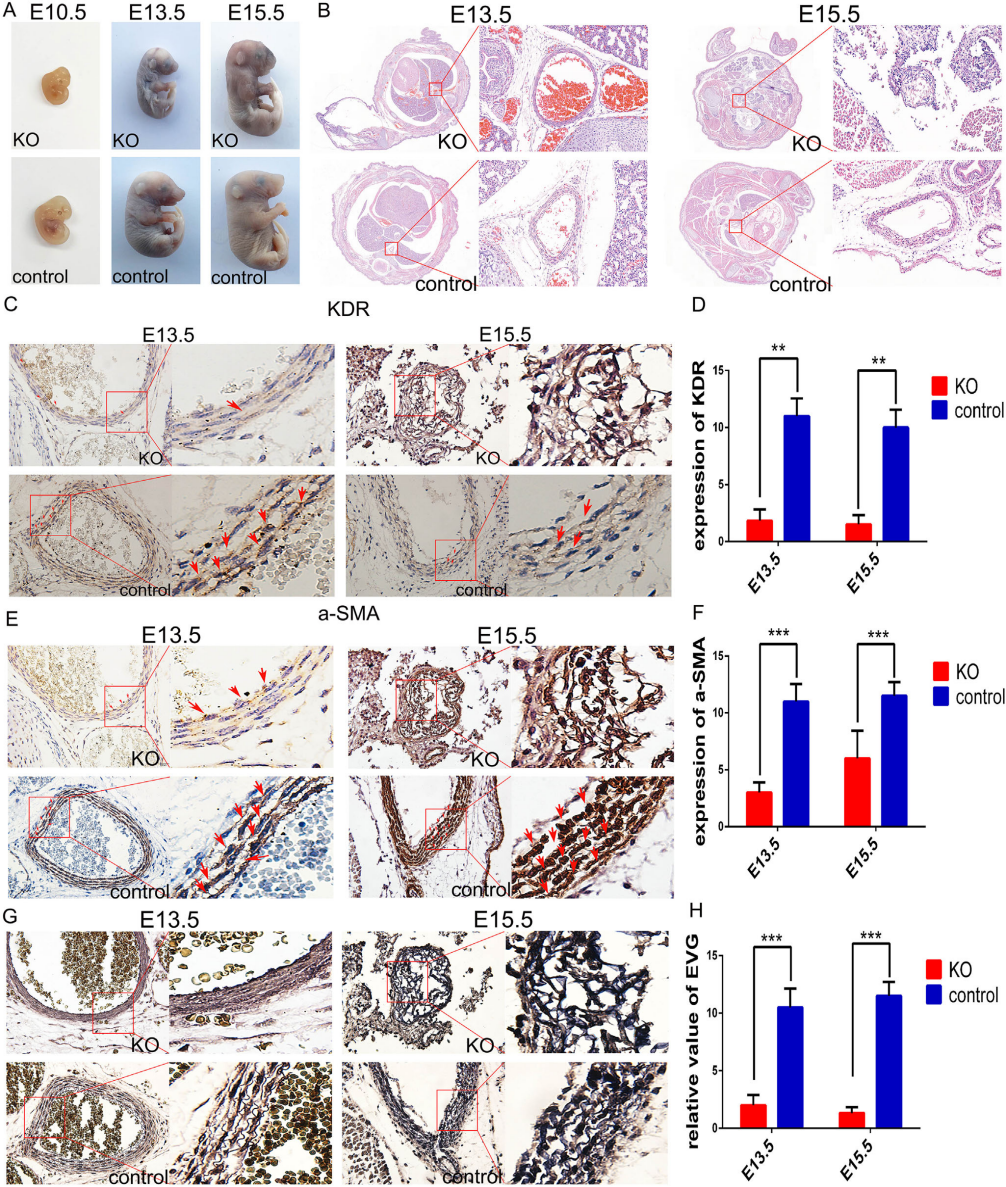
Homozygotes in TFR1 knockout mice die from embryonic stage day 13.5 (A) and are accompanied by abnormalities in the aortic structure (B). A significant decrease in the expression of KDR and a‐SMA in the homozygous aorta of TFR1 knockout mice (C‐F). (G and H) Elastic fibers in the aorta of TFR1 knockout mice were significantly reduced when compared to the normal developing aorta. *N* = 3, values are the mean ± SEM. **p < .01, ***p < .0001 versus tissues from normal developing aorta by *t*‐test analysis of variance

The above‐mentioned animal experiments confirmed that ID can cause AMD. These experiments were designed in acquired iron‐deficient mice, and the mouse aorta has been developed during this period. To investigate the effects of ID on aortic development, TFR1 KO mice were used. We observed that homozygous TFR1 KO mice died on embryonic day 13.5 and had a thinner aorta, while heterozygotes were normal (Figures 7A and 7B). Furthermore, the expression of vascular endothelial growth factor receptor 2 and alpha‐smooth muscle actin was significantly decreased in embryonic TFR1‐/‐ mouse aorta (Figures 7C‐7F). EVG staining showed that elastic fibers in the aorta of TFR1‐/‐ mice at day E13.5 were rare, and almost disappeared (Figures 7G and 7H). These findings suggest that iron is one of the essential elements for the aortic development.

### 4.8 ID induced cytoskeleton destruction and dysfunction of VSMCs in vitro

**FIGURE 8:**
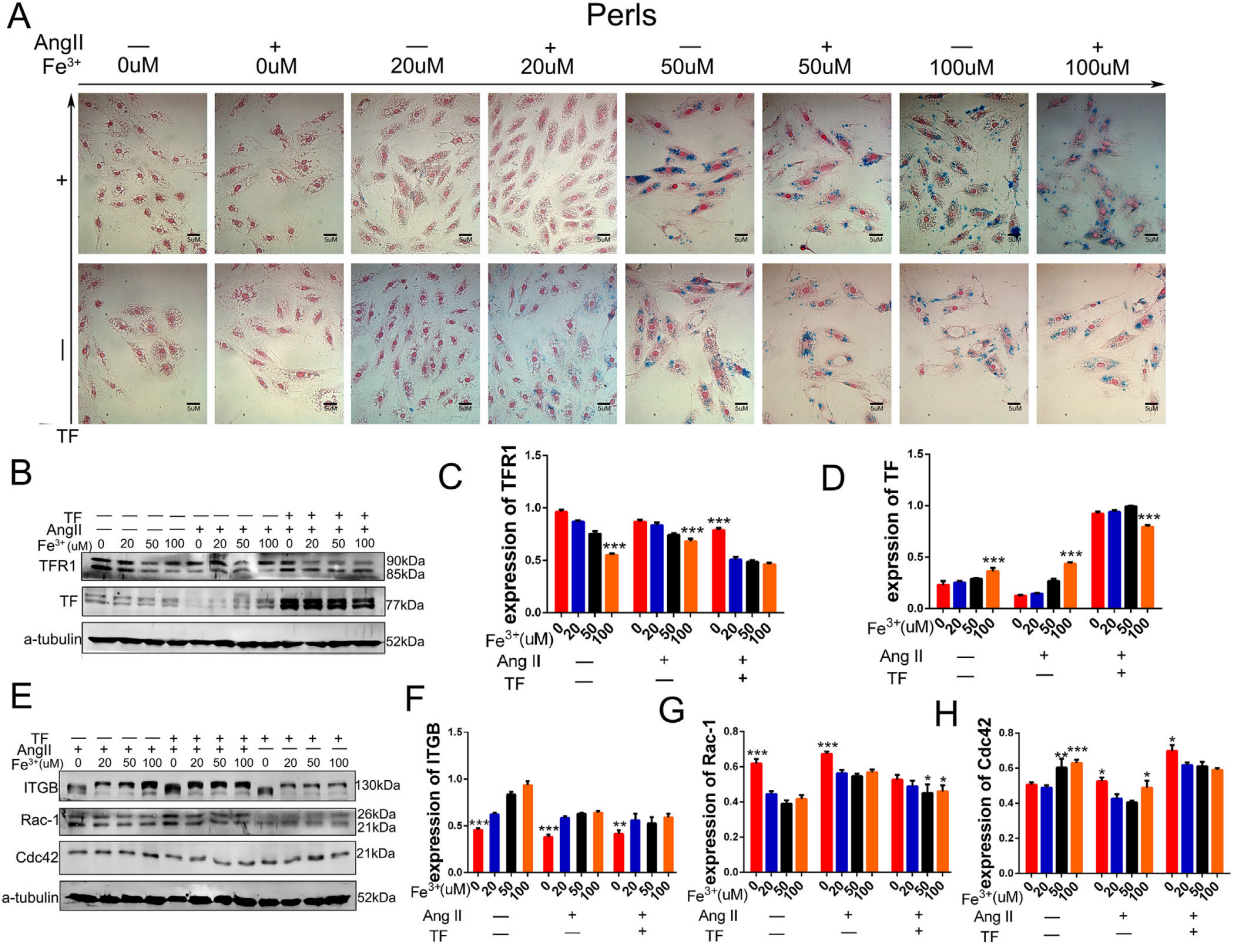
(A) Iron deficiency caused vascular smooth muscle cells to become swollen, and the connections between cells became sparse. Iron supplementation improved this condition, but excessive iron concentration also caused cell damage, and transferrin (TF) assisted more iron into the cell. (B‐D) Iron deficiency increased the expression of TFR1, and the expression of TF in vascular smooth muscle cells (VSMCs) was low. (E‐H) Iron deficiency reduced the expression of cleavage ITGB and Cdc42, and increased the expression of Rac1. *N* = 3, values are the mean ± SEM. *p < .05, **p < .01, ***p < .0001 versus different intervention by two‐way analysis of variance

Animal experiments confirmed that ID can regulate the expression of VSMC cytoskeletal proteins, however, these changes are influenced by multiple factors in vivo. To accurately investigate the effects of ID on the morphology and contractive function of VSMCs, cell experiments were performed in vitro. When cells were iron deficient, they became swollen, the expression of F‐actin reduced, and the gap between cells became larger. With the addition of iron, the cell morphology gradually returned to normal, and the gap between cells reduced. However, when the iron concentration was too high, cells underwent further damaged (Figure 8A, Figure S3A). TF promoted iron transport into cells (Figure 8A, Figure S3B). Moreover, the addition of iron did not affect the osmotic pressure of the medium (Figure 3E). The expression of TF positively correlated with the iron concentration, low iron negative feedback increased the expression of TFR1, and Ang II did not affect iron metabolism (Figures 8B‐8D). Consistent with our expectation, ID reduced the expression of cleaved ITGB and Cdc42, and increased the expression of Rac1 (Figures 8E‐8H). Together, these observations indicate that ID disrupts the cytoskeleton and contractive function of VSMCs.

## 5 DISCUSSION

The main findings of the present study were that low levels of circulating and aortic iron were associated with VSMC dysfunction and aortic instability. ID increased the incidence and severity of AD, as well as the dysregulation of VSMCs, which induced by ID may be achieved through the integrin pathway. Moreover, congenital ID caused vascular developmental disorders.

AD may occur after a tear in the intima layer of the aortic wall, which allows blood to dissect longitudinally through the media, thereby causing a false lumen along the aorta.^22^ There is no effective conservative treatment plan for Stanford type A AD, thus, it is critical to identify the risk factors for the prevention of the disease. Hypertension is a global disease, and with changes in people's lifestyle, the prevalence of hypertension is increasing annually.^23^ Hypertension is an independent risk factor for the prognosis of AD^23^, ^24^ and is associated with significant changes in the mechanical properties of the aortic wall, with more strain‐induced to AD in the presence of hypertension.^25^ In our previous study, we reported patients with AD with a high blood pressure,^4^ and in the present study, we recruited hypertensive patients as a control group for disease‐matching studies to investigate why not all hypertensive patients present with AD.

Iron is like a double‐edged sword. The body needs enough iron to maintain normal physiological functions, but iron provides electrons for oxidative stress, and promotes the generation of ROS.^26^ The mechanisms underlying the detrimental effects of ID in aortic diseases are only just to be explored, and there is controversy about the exact state of iron metabolism in the pathogenesis of aortic diseases. Although several investigators believe that iron deposition in the blood and aortic wall is increased in patients with abdominal AA, the number of cases included in these studies is too less.^14^, ^15^, ^16^, ^19^ When two other articles were included a sufficient sample size, the results were however, it is suggested that iron in patients with abdominal AA is lacking.^17^, ^18^ However, none of these studies indicated a specific correlation between the status of iron metabolism and aortic diseases. In this study, we found that the levels of iron, TF, TS, and TIBC in the circulation of patients with AD and AA were lower compared to that of hypertensive patients. Interestingly, there is no significant difference between the Hb concentration in the blood of patients with aortic disease and the control group. This is because the body will give priority to the production of Hb when using iron, ID anemia occurs when the iron storage in the body is exhausted.^27^, ^28^, ^29^ In addition, we showed that iron deposition and the expression of TF in AD specimens were less than that of organ donors. These findings indicated that there was ID in the circulation and aortic wall of patients with AD/AA. In addition, correlation analysis suggested an inverse association of iron concentration and aortic disease in hypertensive patients. Logistic regression analysis further indicated ID in patients with hypertension as an independent risk factor for aortic disease, especially in AD. Besides, we found that the expression of TFR1 in the aortic wall of patients with AD was significantly higher compared to that of hypertensive patients, and we believe that the expression of TFR1 is increased when ID in the vessel wall during the onset of AD. This was confirmed by our mouse experiments. When mice were fed a low‐iron diet, the iron content in the circulation was lower compared to that of normal‐chow‐fed mice; however, no significant differences were observed in blood pressure between the two groups. After the induction of hypertension with Ang II, the survival time of low‐iron‐fed mice was significantly shortened, the incidence of AD was higher, and the dissection tear was wider. Immunohistochemical staining showed that TF expression decreased, and TFR1 expression increased in the aorta of low‐iron‐fed mice. In addition, embryos from TFR1‐/‐ mice induced smooth muscle cell dysplasia and died in the second trimester (E13.5) due to iron input barriers. These data suggested that ID can indeed promote the formation of AD in hypertensive mice by damaging the vessel wall. The underlying mechanism of this induction remains unclear.

We and others previously demonstrated that normal morphology and function of VSMCs is the basis for maintaining the stability of the arterial wall.^30^, ^31^, ^32^ Dysregulation of VSMCs and the turbulence of junctions between cells are major pathological manifestations of AMD,^4^, ^26^, ^33^, ^34^, ^35^ however, few studies have linked iron metabolism to the regulation of VSMCs. In the present study, we showed that ID caused morphological changes in VSMCs and loosening between cells and cells. Furthermore, in addition to a decrease in phosphorylation of the MLC, ID reduced the expression of F‐actin, integrinβ1, and Cdc42, while increasing the expression of Rac1 both in vivo and in vitro, which are important molecular in cytoskeleton and function, and promote the proliferation and migration of VSMCs, thereby indicating that ID may contribute to the formation of AD by changing the morphology and function of VSMCs.

## 6 STUDY LIMITATIONS

Patients with ID had other comorbidities and used ongoing medication, which may have influenced the status of the aorta. In this study, we showed that the number of preoperative ultrasound‐detectable liver lesions was significantly increased in patients with aortic disease compared to hypertensive patients. However, the decrease in Hb concentration in patients with aortic disease was not significant. In addition, low‐iron‐fed mice, a pure iron‐deficient model, also had lesions of the aortic media but no other symptoms, thereby suggesting that the ID induced AMD. Moreover, several pharmacological compounds of hypertensive patients are known to improve vascular remodeling by various mechanisms.^36^ Most of our patients with aortic disease did not regularly control blood pressure. It may therefore be expected that the medication may counteract the observed negative effect, thus leading to an underestimation of the difference between patients with aortic diseases and hypertension. Interestingly, the serum iron metabolism assay from a subgroup of hypertensive patients who did not receive medical treatment showed no ID, and none of our organ donors had a history of taking antihypertensive drugs. Despite the observations that patients who donated the aorta had AMD, and that ID impaired VSMC function, it cannot be determined to what degree the iron supplement contributed to the repair of the aortic structure in vivo. Only an isolated aortic special ID model permits the identification of the underlying pathophysiological mechanism mediated by ID as it is not feasible to distinguish the effects mediated by ID from those of other lesions in the in vivo setting.

## 7 CONCLUSIONS

Here, we demonstrate a novel disease risk by which ID increases the formation of AMD in patients with hypertension. This effect is triggered by decreased F‐actin and integrin β1‐Cdc42‐Rac1 axis‐derived cytoskeleton and plays a role in the dysregulation of VSMCs during ID. Our findings suggest that ID is an independent risk factor for AMD in patients with hypertension.

## CONFLICT OF INTEREST

The authors declare that there is no conflict of interest that could be perceived as prejudicing the impartiality of the research reported.

## ETHICS APPROVAL AND CONSENT TO PARTICIPATE

All experimental protocols regarding human materials were conducted according to the Declaration of Helsinki and were approved by the ethical committee of Renmin Hospital of Wuhan University (WDRY2015‐K021). All protocols regarding animal studies were conducted in accordance with the guidelines for the management and use of laboratory animals of China (China State Council publication number: 588, revised 2017) and were approved by the Institutional Ethics Committee on Animal Use.

## AUTHOR CONTRIBUTIONS

Bowen Li designed and performed experiments, analyzed data, and wrote the paper. Zhiwei Wang designed the research program. Junmou Hong conceived the idea. Yanjia Che, Ruoshi Chen, Qi Wu, and Junxia Hu were involved in data analysis. Zhipeng Hu, Xiaoping Hu, and Min Zhang participated in manuscript editing. All authors read and approved the final manuscript.

## AVAILABILITY OF DATA AND MATERIALS

The data in the current study are based on public data available, and some data used during the study appear in the supplementary material and figure legends. All data that support the findings of this study are available from the corresponding author upon reasonable request.

## Supporting information

Supporting Information

Supporting Information

Supporting Information

Supporting Information

Supporting Information

Supporting Information

Supporting Information

Supporting Information

Supporting Information

Supporting Information

Supporting Information

Supporting Information

Supporting Information
