# Supplementary material for: Iron deficiency promotes aortic medial degeneration via destructing cytoskeleton of vascular smooth muscle cells
Source: Clin Transl Med. 2021 Jan 13;11(1):e276. doi: 10.1002/ctm2.276 (PMC7805404; doi:10.1002/ctm2.276)
Supplement: Supplementary file 1 — Supporting Information [file CTM2-11-e276-s001.doc]

Multivariate regression analysis of iron concentration and correlation between different types of aortic diseases

| Aortic Disease |  | β | SE | Wald | Df | p-value | OR | 95%CI |
| --- | --- | --- | --- | --- | --- | --- | --- | --- |
| Type A | Intercept | 28.230 | 5.009 | 31.765 | 1 | 0.000 |  |  |
|  | Age | -0.034 | 0.036 | 0.887 | 1 | 0.346 | 0.967 | 0.902-1.037 |
|  | CRP | -0.024 | 0.016 | 2.273 | 1 | 0.132 | 0.976 | 0.946-1.007 |
|  | PT | -0.508 | 0.176 | 8.315 | 1 | 0.004** | 0.602 | 0.426-0.850 |
|  | D-Dimer | -0.795 | 0.168 | 22.300 | 1 | 0.000*** | 0.451 | 0.324-0.628 |
|  | Fe^3+^ | 0.257 | 0.056 | 21.213 | 1 | 0.000*** | 1.294 | 1.159-1.443 |
|  | FER | -0.004 | 0.002 | 3.724 | 1 | 0.054 | 0.996 | 0.993-1.000 |
|  | BMI | -0.856 | 0.121 | 50.088 | 1 | 0.000*** | 0.425 | 0.335-0.539 |
|  | Male | -1.984 | 1.067 | 3.461 | 1 | 0.063 | 0.137 | 0.017-1.112 |
|  | Female | 0^b^ | . | . | 0 | . | . |  |
|  | No alcohol | -0.716 | 1.518 | 0.222 | 1 | 0.637 | 0.489 | 0.025-9.577 |
|  | Alcohol 1-100 g/d | 3.367 | 0.908 | 13.740 | 1 | 0.000*** | 29.005 | 4.889-172.097 |
|  | Alcohol 101-150 g/d | 1.877 | 1.007 | 3.473 | 1 | 0.062 | 6.535 | 0.907-47.068 |
|  | Alcohol>150g/d | 0^b^ | . | . | 0 | . | . |  |
| Type B | Intercept | 1.867 | 2.531 | 0.545 | 1 | 0.461 |  |  |
|  | Age | 0.186 | 0.019 | 94.503 | 1 | 0.000*** | 1.205 | 1.160-1.251 |
|  | CRP | -0.004 | 0.005 | 0.703 | 1 | 0.402 | 0.996 | 0.986-1.005 |
|  | PT | -0.211 | 0.129 | 2.672 | 1 | 0.102 | 0.809 | 0.628-1.043 |
|  | D-Dimer | -0.144 | 0.035 | 17.080 | 1 | 0.000*** | 0.866 | 0.809-0.927 |
|  | Fe^3+^ | 0.097 | 0.031 | 9.721 | 1 | 0.002** | 1.101 | 1.037-1.170 |
|  | FER | -0.002 | 0.001 | 3.459 | 1 | 0.063 | 0.998 | 0.995-1.000 |
|  | BMI | -0.454 | 0.047 | 93.541 | 1 | 0.000*** | 0.635 | 0.579-0.696 |
|  | Male | 1.854 | 0.877 | 4.467 | 1 | 0.035* | 6.387 | 1.144-35.648 |
|  | Female | 0^b^ | . | . | 0 | . | . |  |
|  | No alcohol | -0.412 | 0.951 | 0.188 | 1 | 0.665 | 0.662 | 0.103-4.271 |
|  | Alcohol 1-100 g/d | 1.089 | 0.464 | 5.501 | 1 | 0.019* | 2.972 | 1.196-7.386 |
|  | Alcohol 101-150 g/d | -0.776 | 0.558 | 1.932 | 1 | 0.165 | 0.460 | 0.154-1.374 |
|  | Alcohol>150g/d | 0^b^ | . | . | 0 | . | . |  |
| AA | Intercept | 5.686 | 1.539 | 13.657 | 1 | 0.000*** |  |  |
|  | Age | 0.061 | 0.010 | 36.649 | 1 | 0.000*** | 1.063 | 1.042-1.084 |
|  | CRP | 0.000 | 0.003 | 0.000 | 1 | 0.983 | 1.000 | 0.994-1.006 |
|  | PT | -0.157 | 0.077 | 4.150 | 1 | 0.042* | 0.855 | 0.735-0.994 |
|  | D-Dimer | -0.062 | 0.018 | 11.585 | 1 | 0.001** | 0.940 | 0.907-0.974 |
|  | Fe^3+^ | 0.037 | 0.020 | 3.366 | 1 | 0.067 | 1.037 | 0.998-1.079 |
|  | FER | -0.001 | 0.001 | 3.381 | 1 | 0.066 | 0.999 | 0.998-1.000 |
|  | BMI | -0.232 | 0.030 | 60.223 | 1 | 0.000*** | 0.793 | 0.748-0.841 |
|  | Male | 0.319 | 0.503 | 0.403 | 1 | 0.526 | 1.376 | 0.513-3.688 |
|  | Female | 0^b^ | . | . | 0 | . | . |  |
|  | No alcohol | -1.929 | 0.609 | 10.028 | 1 | 0.002** | 0.145 | 0.044-0.479 |
|  | Alcohol 1-100 g/d | 1.039 | 0.309 | 11.275 | 1 | 0.001** | 2.826 | 1.541-5.184 |
|  | Alcohol 101-150 g/d | 0.158 | 0.331 | 0.228 | 1 | 0.633 | 1.171 | 0.613-2.238 |
|  | Alcohol>150g/d | 0^b^ | . | . | 0 | . | . |  |

a. Reference group for hypertension group b. This parameter is set to zero because this is redundant.

CRP=C-reaction protein; PT=Prothrombin time; FER=Ferritin; BMI=Body mass index.
