# Supplementary material for: Iron deficiency promotes aortic medial degeneration via destructing cytoskeleton of vascular smooth muscle cells
Source: Clin Transl Med. 2021 Jan 13;11(1):e276. doi: 10.1002/ctm2.276 (PMC7805404; doi:10.1002/ctm2.276)
Supplement: Supplementary file 2 — Supporting Information [file CTM2-11-e276-s002.doc]

Characteristics of patients with aortic diseases

|  |  | Male group | Female group | P-value |
| --- | --- | --- | --- | --- |
| Type A AD | Quantity (n) | 48 | 18 | <0.0001*** |
|  | Age (years) | 50.73±1.54 | 56.39±2.37 | 0.0555 |
|  | BMI | 31.87±0.65 | 31.35±1.02 | 0.6792 |
|  | Comorbidities (n) |  |  |  |
|  | Hypertension | 48 | 18 | 1.0000 |
|  | Genetic disorders | untested | untested |  |
|  | Diabetes mellitus | 2 | 0 | 1.0000 |
|  | Trauma | 0 | 0 | 1.0000 |
|  | Atherosclerosis | 5 | 2 | 1.0000 |
|  | Gastrointestinal disease | 4 | 4 | 0.1991 |
|  | Neurodegenerative disease | 0 | 0 | 1.0000 |
|  | Iron concentration in serum | 7.87±0.81 | 5.38±0.71 | 0.0831 |
| Type B AD | Quantity (n) | 77 | 10 | <0.0001*** |
|  | Age (years) | 57.55±1.39 | 61.80±4.69 | 0.3165 |
|  | BMI | 28.32±0.53 | 27.30±1.63 | 0.5207 |
|  | Cause of disease(n) |  |  |  |
|  | Hypertension | 77 | 10 | 1.0000 |
|  | Genetic disorders | untested | untested |  |
|  | Diabetes mellitus | 3 | 2 | 0.0990 |
|  | Trauma | 0 | 0 | 1.0000 |
|  | Atherosclerosis | 12 | 2 | 0.6603 |
|  | Gastrointestinal disease | 0 | 0 | 1.0000 |
|  | Neurodegenerative disease | 0 | 0 | 1.0000 |
|  | Iron concentration in serum | 9.46±0.75 | 8.03±1.92 | 0.5152 |
| Aortic aneurysm | Quantity (n) | 39 | 8 | <0.0001*** |
|  | Age (years) | 69.79±1.44 | 78.00±2.28 | 0.0184** |
|  | BMI | 24.49±0.52 | 24.88±1.20 | 0.7617 |
|  | Cause of disease(n) |  |  |  |
|  | Hypertension | 22 | 4 | 1.0000 |
|  | Genetic disorders | untested | untested |  |
|  | Diabetes mellitus | 5 | 6 | 0.0010 |
|  | Trauma | 0 | 0 | 1.0000 |
|  | Atherosclerosis | 37 | 8 | 1.0000 |
|  | Gastrointestinal disease | 4 | 6 | 0.0005*** |
|  | Neurodegenerative disease | 0 | 0 | 1.0000 |
|  | Iron concentration in serum | 11.60±0.87 | 9.38±1.73 | 0.2908 |

BMI=Body mass index.
