# Supplementary material for: Iron deficiency promotes aortic medial degeneration via destructing cytoskeleton of vascular smooth muscle cells
Source: Clin Transl Med. 2021 Jan 13;11(1):e276. doi: 10.1002/ctm2.276 (PMC7805404; doi:10.1002/ctm2.276)
Supplement: Supplementary file 3 — Supporting Information [file CTM2-11-e276-s003.doc]

post hoc test of analysis of serum concentration of Fe^3+^ (one-way ANOVA)

| Number of families | 1 |  |  |  |  |
| --- | --- | --- | --- | --- | --- |
| Number of comparisons per family | 6 |  |  |  |  |
| Alpha | 0.05 |  |  |  |  |
|  |  |  |  |  |  |
| Tukey’s multiple comparisons test | Mean Diff. | 95% CI of diff. | Significant? | Summary | Adjusted P Value |
|  |  |  |  |  |  |
| Type A AD vs. Type B AD | -2.147 | -4.534 to 0.2396 | No | Ns | 0.0948 |
| Type A AD vs. AA | -4.062 | -6.832 to -1.291 | Yes | ** | 0.0011 |
| Type A AD vs. Control | -9.748 | -12.35 to -7.141 | Yes | **** | <0.0001 |
| Type B AD vs. AA | -1.915 | -4.532 to 0.7026 | No | Ns | 0.2341 |
| Type B AD vs. Control | -7.601 | -10.04 to -5.158 | Yes | **** | <0.0001 |
| AA vs. Control | -5.686 | -8.505 to -2.867 | yes | **** | <0.0001 |

AD=aortic dissection, AA=aortic aneurysm

post hoc test of analysis of serum concentration of TIBC (one-way ANOVA)

AD=aortic dissection, AA=aortic aneurysm

| Number of families | 1 |  |  |  |  |
| --- | --- | --- | --- | --- | --- |
| Number of comparisons per family | 6 |  |  |  |  |
| Alpha | 0.05 |  |  |  |  |
|  |  |  |  |  |  |
| Tukey’s multiple comparisons test | Mean Diff. | 95% CI of diff. | Significant? | Summary | Adjusted P Value |
|  |  |  |  |  |  |
| Type A AD vs. Type B AD | -0.5321 | -3.647 to 2.583 | No | Ns | 0.9711 |
| Type A AD vs. AA | -1.751 | -5.408 to 1.905 | No | NS | 0.6029 |
| Type A AD vs. Control | -11.29 | -14.69 to -7.896 | Yes | **** | <0.0001 |
| Type B AD vs. AA | -1.219 | -4.668 to 2.229 | No | Ns | 0.7972 |
| Type B AD vs. Control | -10.76 | -13.94 to -7.588 | Yes | **** | <0.0001 |
| AA vs. Control | -9.544 | -13.25 to -5.837 | Yes | **** | <0.0001 |

post hoc test of analysis of serum concentration of TS (one-way ANOVA)

| Number of families | 1 |  |  |  |  |
| --- | --- | --- | --- | --- | --- |
| Number of comparisons per family | 6 |  |  |  |  |
| Alpha | 0.05 |  |  |  |  |
|  |  |  |  |  |  |
| Tukey’s multiple comparisons test | Mean Diff. | 95% CI of diff. | Significant? | Summary | Adjusted P Value |
|  |  |  |  |  |  |
| Type A AD vs. Type B AD | -3.945 | -9.093 to 1.202 | No | Ns | 0.1975 |
| Type A AD vs. AA | -8.338 | -14.38 to -2.296 | Yes | ** | 0.0024 |
| Type A AD vs. Control | -14.84 | -20.43 to -9.247 | Yes | **** | <0.0001 |
| Type B AD vs. AA | -4.393 | -10.09 to 1.306 | No | Ns | 0.1931 |
| Type B AD vs. Control | -10.90 | -16.12 to -5.675 | Yes | **** | <0.0001 |
| AA vs. Control | -6.502 | -12.61 to -0.3981 | Yes | * | 0.0318 |

AD=aortic dissection, AA=aortic aneurysm

post hoc test of analysis of serum concentration of TF (one-way ANOVA)

| Number of families | 1 |  |  |  |  |
| --- | --- | --- | --- | --- | --- |
| Number of comparisons per family | 6 |  |  |  |  |
| Alpha | 0.05 |  |  |  |  |
|  |  |  |  |  |  |
| Tukey’s multiple comparisons test | Mean Diff. | 95% CI of diff. | Significant? | Summary | Adjusted P Value |
|  |  |  |  |  |  |
| Type A AD vs. Type B AD | -0.1021 | -0.2679 to 0.0636 | No | Ns | 0.3840 |
| Type A AD vs. AA | -0.1440 | -0.3378 to 0.0499 | No | ns | 0.2218 |
| Type A AD vs. Control | -0.4607 | -0.6404 to -0.281 | Yes | **** | <0.0001 |
| Type B AD vs. AA | -0.0418 | -0.2228 to 0.1391 | No | Ns | 0.9326 |
| Type B AD vs. Control | -0.3585 | -0.5243 to -0.193 | Yes | **** | <0.0001 |
| AA vs. Control | -0.3167 | -0.5105 to -0.123 | Yes | *** | 0.0002 |

AD=aortic dissection, AA=aortic aneurysm

post hoc test of analysis of serum concentration of sTFR (one-way ANOVA)

| Number of families | 1 |  |  |  |  |
| --- | --- | --- | --- | --- | --- |
| Number of comparisons per family | 6 |  |  |  |  |
| Alpha | 0.05 |  |  |  |  |
|  |  |  |  |  |  |
| Tukey’s multiple comparisons test | Mean Diff. | 95% CI of diff. | Significant? | Summary | Adjusted P Value |
|  |  |  |  |  |  |
| Type A AD vs. Type B AD | -0.2320 | -2.441 to 1.976 | No | Ns | 0.9930 |
| Type A AD vs. AA | 0.2788 | -2.313 to 2.871 | No | ns | 0.9925 |
| Type A AD vs. Control | -1.352 | -3.752 to 1.048 | No | ns | 0.4650 |
| Type B AD vs. AA | 0.5108 | -1.934 to 2.956 | No | Ns | 0.9490 |
| Type B AD vs. Control | -1.120 | -3.360 to 1.119 | No | ns | 0.5679 |
| AA vs. Control | -1.631 | -4.250 to 0.9878 | No | ns | 0.3745 |

AD=aortic dissection, AA=aortic aneurysm

post hoc test of analysis of serum concentration of SF (one-way ANOVA)

| Number of families | 1 |  |  |  |  |
| --- | --- | --- | --- | --- | --- |
| Number of comparisons per family | 6 |  |  |  |  |
| Alpha | 0.05 |  |  |  |  |
|  |  |  |  |  |  |
| Tukey’s multiple comparisons test | Mean Diff. | 95% CI of diff. | Significant? | Summary | Adjusted P Value |
|  |  |  |  |  |  |
| Type A AD vs. Type B AD | 57.05 | -29.82 to 143.9 | No | ns | 0.3267 |
| Type A AD vs. AA | 107.1 | 5.138 to 209.1 | Yes | * | 0.0353 |
| Type A AD vs. Control | 177.1 | 82.73 to 271.5 | Yes | **** | "< 0.0001" |
| Type B AD vs. AA | 50.06 | -46.11 to 146.2 | No | ns | 0.5344 |
| Type B AD vs. Control | 120.1 | 31.99 to 208.2 | Yes | ** | 0.0028 |
| AA vs. Control | 70.02 | -32.99 to 173.0 | No | ns | 0.2962 |

AD=aortic dissection, AA=aortic aneurysm

post hoc test of analysis of serum concentration of SFA (one-way ANOVA)

| Number of families | 1 |  |  |  |  |
| --- | --- | --- | --- | --- | --- |
| Number of comparisons per family | 6 |  |  |  |  |
| Alpha | 0.05 |  |  |  |  |
|  |  |  |  |  |  |
| Tukey’s multiple comparisons test | Mean Diff. | 95% CI of diff. | Significant? | Summary | Adjusted P Value |
|  |  |  |  |  |  |
| Type A AD vs. Type B AD | 2.008 | 0.2535 to 3.762 | Yes | * | 0.0176 |
| Type A AD vs. AA | 1.573 | -0.4866 to 3.632 | No | ns | 0.2002 |
| Type A AD vs. Control | -2.563 | -4.469 to -0.6567 | Yes | ** | 0.0033 |
| Type B AD vs. AA | -0.4352 | -2.377 to 1.507 | No | ns | 0.9382 |
| Type B AD vs. Control | -4.571 | -6.350 to -2.792 | Yes | **** | "< 0.0001" |
| AA vs. Control | -4.136 | -6.216 to -2.055 | Yes | **** | "< 0.0001" |

AD=aortic dissection, AA=aortic aneurysm

post hoc test of analysis of serum concentration of ViB12 (one-way ANOVA)

| Number of families | 1 |  |  |  |  |
| --- | --- | --- | --- | --- | --- |
| Number of comparisons per family | 6 |  |  |  |  |
| Alpha | 0.05 |  |  |  |  |
|  |  |  |  |  |  |
| Tukey’s multiple comparisons test | Mean Diff. | 95% CI of diff. | Significant? | Summary | Adjusted P Value |
|  |  |  |  |  |  |
| Type A AD vs. Type B AD | 16.53 | -86.77 to 119.8 | No | ns | 0.9761 |
| Type A AD vs. AA | 129.8 | 8.510 to 251.0 | Yes | * | 0.0307 |
| Type A AD vs. Control | 51.39 | -60.85 to 163.6 | No | ns | 0.6375 |
| Type B AD vs. AA | 113.2 | -1.120 to 227.6 | No | ns | 0.0534 |
| Type B AD vs. Control | 34.86 | -69.89 to 139.6 | No | ns | 0.8250 |
| AA vs. Control | -78.37 | -200.9 to 44.12 | No | ns | 0.3501 |

AD=aortic dissection, AA=aortic aneurysm

post hoc test of analysis of serum concentration of Hb (one-way ANOVA)

| Number of families | 1 |  |  |  |  |
| --- | --- | --- | --- | --- | --- |
| Number of comparisons per family | 6 |  |  |  |  |
| Alpha | 0.05 |  |  |  |  |
|  |  |  |  |  |  |
| Tukey’s multiple comparisons test | Mean Diff. | 95% CI of diff. | Significant? | Summary | Adjusted P Value |
|  |  |  |  |  |  |
| Type A AD vs. Type B AD | -2.410 | -10.01 to 5.188 | No | ns | 0.8448 |
| Type A AD vs. AA | 1.656 | -7.208 to 10.52 | No | ns | 0.9628 |
| Type A AD vs. Control | -4.091 | -12.35 to 4.165 | No | ns | 0.5756 |
| Type B AD vs. AA | 4.066 | -4.287 to 12.42 | No | ns | 0.5900 |
| Type B AD vs. Control | -1.681 | -9.386 to 6.024 | No | ns | 0.9426 |
| AA vs. Control | -5.747 | -14.70 to 3.209 | No | ns | 0.3474 |

AD=aortic dissection, AA=aortic aneurysm

post hoc test of analysis of iron deposition of mice aorta (one-way ANOVA)

| Number of families | 1 |  |  |  |  |
| --- | --- | --- | --- | --- | --- |
| Number of comparisons per family | 6 |  |  |  |  |
| Alpha | 0.05 |  |  |  |  |
|  |  |  |  |  |  |
| Tukey’s multiple comparisons test | Mean Diff. | 95% CI of diff. | Significant? | Summary | Adjusted P Value |
|  |  |  |  |  |  |
| " control vs. AngII " | 1.167 | "-1.447 to 3.781" | No | ns | 0.6042 |
| " control vs. ID" | 6.000 | "3.386 to 8.614" | Yes | **** | "< 0.0001" |
| " control vs. ID+AngII" | 6.500 | "3.886 to 9.114" | Yes | **** | "< 0.0001" |
| " AngII vs. ID" | 4.833 | "2.219 to 7.447" | Yes | *** | 0.0002 |
| " AngII vs. ID+AngII" | 5.333 | "2.719 to 7.947" | Yes | **** | "< 0.0001" |
| " ID vs. ID+AngII" | 0.5000 | "-2.114 to 3.114" | No | ns | 0.9494 |

Ang II= angiotensin II, ID= iron deficiency

post hoc test of analysis of TF expression of mice aorta (one-way ANOVA)

| Number of families | 1 |  |  |  |  |
| --- | --- | --- | --- | --- | --- |
| Number of comparisons per family | 6 |  |  |  |  |
| Alpha | 0.05 |  |  |  |  |
|  |  |  |  |  |  |
| Tukey’s multiple comparisons test | Mean Diff. | 95% CI of diff. | Significant? | Summary | Adjusted P Value |
|  |  |  |  |  |  |
| " control vs. AngII " | 1.167 | "-1.128 to 3.461" | No | ns | 0.5003 |
| " control vs. ID" | 6.667 | "4.372 to 8.961" | Yes | **** | "< 0.0001" |
| " control vs. ID+AngII" | 7.500 | "5.205 to 9.795" | Yes | **** | "< 0.0001" |
| " AngII vs. ID" | 5.500 | "3.205 to 7.795" | Yes | **** | "< 0.0001" |
| " AngII vs. ID+AngII" | 6.333 | "4.039 to 8.628" | Yes | **** | "< 0.0001" |
| " ID vs. ID+AngII" | 0.8333 | "-1.461 to 3.128" | No | ns | 0.7419 |

Ang II= angiotensin II, ID= iron deficiency

post hoc test of analysis of TFR1 expression of mice aorta (one-way ANOVA)

| Number of families | 1 |  |  |  |  |
| --- | --- | --- | --- | --- | --- |
| Number of comparisons per family | 6 |  |  |  |  |
| Alpha | 0.05 |  |  |  |  |
|  |  |  |  |  |  |
| Tukey’s multiple comparisons test | Mean Diff. | 95% CI of diff. | Significant? | Summary | Adjusted P Value |
|  |  |  |  |  |  |
| " control vs. AngII " | -1.167 | "-3.895 to 1.561" | No | ns | 0.6357 |
| " control vs. ID" | -5.000 | "-7.728 to -2.272" | Yes | *** | 0.0003 |
| " control vs. ID+AngII" | -5.500 | "-8.228 to -2.772" | Yes | **** | "< 0.0001" |
| " AngII vs. ID" | -3.833 | "-6.561 to -1.105" | Yes | ** | 0.0042 |
| " AngII vs. ID+AngII" | -4.333 | "-7.061 to -1.605" | Yes | ** | 0.0013 |
| " ID vs. ID+AngII" | -0.5000 | "-3.228 to 2.228" | No | ns | 0.9550 |

Ang II= angiotensin II, ID= iron deficiency

post hoc test of analysis of IGTB expression of mice aorta (one-way ANOVA)

| Number of families | 1 |  |  |  |  |
| --- | --- | --- | --- | --- | --- |
| Number of comparisons per family | 6 |  |  |  |  |
| Alpha | 0.05 |  |  |  |  |
|  |  |  |  |  |  |
| Tukey’s multiple comparisons test | Mean Diff. | 95% CI of diff. | Significant? | Summary | Adjusted P Value |
|  |  |  |  |  |  |
| " control vs. AngII " | 4.167 | "1.797 to 6.536" | Yes | *** | 0.0004 |
| " control vs. ID" | 4.667 | "2.297 to 7.036" | Yes | *** | 0.0001 |
| " control vs. ID+AngII" | 6.833 | "4.464 to 9.203" | Yes | **** | "< 0.0001" |
| " AngII vs. ID" | 0.5000 | "-1.869 to 2.869" | No | ns | 0.9337 |
| " AngII vs. ID+AngII" | 2.667 | "0.2972 to 5.036" | Yes | * | 0.0239 |
| " ID vs. ID+AngII" | 2.167 | "-0.2028 to 4.536" | No | ns | 0.0807 |

Ang II= angiotensin II, ID= iron deficiency

post hoc test of analysis of Cdc42 expression of mice aorta (one-way ANOVA)

| Number of families | 1 |  |  |  |  |
| --- | --- | --- | --- | --- | --- |
| Number of comparisons per family | 6 |  |  |  |  |
| Alpha | 0.05 |  |  |  |  |
|  |  |  |  |  |  |
| Tukey’s multiple comparisons test | Mean Diff. | 95% CI of diff. | Significant? | Summary | Adjusted P Value |
|  |  |  |  |  |  |
| " control vs. AngII " | 6.167 | "3.872 to 8.461" | Yes | **** | "< 0.0001" |
| " control vs. ID" | 6.000 | "3.705 to 8.295" | Yes | **** | "< 0.0001" |
| " control vs. ID+AngII" | 6.167 | "3.872 to 8.461" | Yes | **** | "< 0.0001" |
| " AngII vs. ID" | -0.1667 | "-2.461 to 2.128" | No | ns | 0.9969 |
| " AngII vs. ID+AngII" | 0.0 | "-2.295 to 2.295" | No | ns | "> 0.9999" |
| " ID vs. ID+AngII" | 0.1667 | "-2.128 to 2.461" | No | ns | 0.9969 |

Ang II= angiotensin II, ID= iron deficiency

post hoc test of analysis of Rac-1 expression of mice aorta (one-way ANOVA)

| Number of families | 1 |  |  |  |  |
| --- | --- | --- | --- | --- | --- |
| Number of comparisons per family | 6 |  |  |  |  |
| Alpha | 0.05 |  |  |  |  |
|  |  |  |  |  |  |
| Tukey’s multiple comparisons test | Mean Diff. | 95% CI of diff. | Significant? | Summary | Adjusted P Value |
|  |  |  |  |  |  |
| " control vs. AngII " | -4.000 | "-6.826 to -1.174" | Yes | ** | 0.0039 |
| " control vs. ID" | -3.667 | "-6.493 to -0.8406" | Yes | ** | 0.0083 |
| " control vs. ID+AngII" | -6.167 | "-8.993 to -3.341" | Yes | **** | "< 0.0001" |
| " AngII vs. ID" | 0.3333 | "-2.493 to 3.159" | No | ns | 0.9872 |
| " AngII vs. ID+AngII" | -2.167 | "-4.993 to 0.6594" | No | ns | 0.1730 |
| " ID vs. ID+AngII" | -2.500 | "-5.326 to 0.3260" | No | ns | 0.0947 |

Ang II= angiotensin II, ID= iron deficiency

post hoc test of analysis of TFR1 expression of HASMCs (two-way ANOVA)

| "Number of families" | 3 |  |  |  |  |
| --- | --- | --- | --- | --- | --- |
| "Number of comparisons per family" | 6 |  |  |  |  |
| Alpha | 0.05 |  |  |  |  |
|  |  |  |  |  |  |
| "Tukey's multiple comparisons test" | Mean Diff. | 95% CI of diff. | Significant? | Summary | Adjusted P Value |
|  |  |  |  |  |  |
| Blank |  |  |  |  |  |
| 0 vs. 20 | 0.09442 | 0.05253 to 0.1363 | Yes | **** | "< 0.0001" |
| 0 vs. 50 | 0.2121 | 0.1702 to 0.2540 | Yes | **** | "< 0.0001" |
| 0 vs. 100 | 0.4134 | 0.3715 to 0.4553 | Yes | **** | "< 0.0001" |
| 20 vs. 50 | 0.1176 | 0.07575 to 0.1595 | Yes | **** | "< 0.0001" |
| 20 vs. 100 | 0.3190 | 0.2771 to 0.3608 | Yes | **** | "< 0.0001" |
| 50 vs. 100 | 0.2013 | 0.1594 to 0.2432 | Yes | **** | "< 0.0001" |
|  |  |  |  |  |  |
| AngII |  |  |  |  |  |
| 0 vs. 20 | 0.03342 | -0.008472 to 0.07532 | No | ns | 0.1464 |
| 0 vs. 50 | 0.1259 | 0.08400 to 0.1678 | Yes | **** | < 0.0001 |
| 0 vs. 100 | 0.1864 | 0.1445 to 0.2283 | Yes | **** | < 0.0001 |
| 20 vs. 50 | 0.09247 | 0.05058 to 0.1344 | Yes | **** | < 0.0001 |
| 20 vs. 100 | 0.1530 | 0.1111 to 0.1949 | Yes | **** | < 0.0001 |
| 50 vs. 100 | 0.06052 | 0.01862 to 0.1024 | Yes | ** | 0.0036 |
|  |  |  |  |  |  |
| TF+Ang II |  |  |  |  |  |
| 0 vs. 20 | 0.2805 | 0.2386 to 0.3224 | Yes | **** | < 0.0001 |
| 0 vs. 50 | 0.3023 | 0.2604 to 0.3442 | Yes | **** | < 0.0001 |
| 0 vs. 100 | 0.3265 | 0.2846 to 0.3684 | Yes | **** | < 0.0001 |
| 20 vs. 50 | 0.02184 | -0.02006 to 0.06374 | No | ns | 0.4731 |
| 20 vs. 100 | 0.04597 | 0.004076 to 0.08787 | Yes | * | 0.0287 |
| 50 vs. 100 | 0.02413 | -0.01776 to 0.06603 | No | ns | 0.3887 |

Ang II= angiotensin II, TF= exogenous transferrin

post hoc test of analysis of TF expression of HASMCs (two-way ANOVA)

| "Number of families" | 3 |  |  |  |  |
| --- | --- | --- | --- | --- | --- |
| "Number of comparisons per family" | 6 |  |  |  |  |
| Alpha | 0.05 |  |  |  |  |
|  |  |  |  |  |  |
| "Tukey's multiple comparisons test" | Mean Diff. | 95% CI of diff. | Significant? | Summary | Adjusted P Value |
|  |  |  |  |  |  |
| Blank |  |  |  |  |  |
| 0 vs. 20 | -0.02204 | -0.06130 to 0.01722 | No | ns | 0.4104 |
| 0 vs. 50 | -0.05933 | -0.09858 to -0.02007 | Yes | ** | 0.0024 |
| 0 vs. 100 | -0.1322 | "-0.1714 to -0.09293" | Yes | **** | < 0.0001 |
| 20 vs. 50 | -0.03729 | -0.07654 to 0.001971 | No | ns | 0.0661 |
| 20 vs. 100 | -0.1101 | -0.1494 to -0.07089 | Yes | **** | < 0.0001 |
| 50 vs. 100 | -0.07286 | -0.1121 to -0.03360 | Yes | *** | 0.0003 |
|  |  |  |  |  |  |
| AngII |  |  |  |  |  |
| 0 vs. 20 | -0.01840 | -0.05766 to 0.02086 | No | ns | 0.5599 |
| 0 vs. 50 | -0.1406 | -0.1798 to -0.1013 | Yes | **** | < 0.0001 |
| 0 vs. 100 | -0.3102 | -0.3495 to -0.2710 | Yes | **** | < 0.0001 |
| 20 vs. 50 | -0.1222 | -0.1614 to -0.08292 | Yes | **** | < 0.0001 |
| 20 vs. 100 | -0.2918 | -0.3311 to -0.2526 | Yes | **** | < 0.0001 |
| 50 vs. 100 | -0.1696 | -0.2089 to -0.1304 | Yes | **** | < 0.0001 |
|  |  |  |  |  |  |
| TF+Ang II |  |  |  |  |  |
| 0 vs. 20 | -0.01622 | -0.05548 to 0.02304 | No | ns | 0.6539 |
| 0 vs. 50 | -0.06951 | -0.1088 to -0.03026 | Yes | *** | 0.0005 |
| 0 vs. 100 | 0.1273 | 0.08808 to 0.1666 | Yes | **** | < 0.0001 |
| 20 vs. 50 | -0.05329 | -0.09255 to -0.01403 | Yes | ** | 0.0060 |
| 20 vs. 100 | 0.1436 | 0.1043 to 0.1828 | Yes | **** | < 0.0001 |
| 50 vs. 100 | 0.1969 | 0.1576 to 0.2361 | Yes | **** | < 0.0001 |

Ang II= angiotensin II, TF= exogenous transferrin

post hoc test of analysis of IGTB expression of HASMCs (two-way ANOVA)

| "Number of families" | 3 |  |  |  |  |
| --- | --- | --- | --- | --- | --- |
| "Number of comparisons per family" | 6 |  |  |  |  |
| Alpha | 0.05 |  |  |  |  |
|  |  |  |  |  |  |
| "Tukey's multiple comparisons test" | Mean Diff. | 95% CI of diff. | Significant? | Summary | Adjusted P Value |
|  |  |  |  |  |  |
| Blank |  |  |  |  |  |
| 0 vs. 20 | -0.1677 | -0.2519 to -0.08346 | Yes | **** | < 0.0001 |
| 0 vs. 50 | -0.3787 | -0.4630 to -0.2945 | Yes | **** | < 0.0001 |
| 0 vs. 100 | -0.4819 | -0.5661 to -0.3977 | Yes | **** | < 0.0001 |
| 20 vs. 50 | -0.2111 | -0.2953 to -0.1268 | Yes | **** | < 0.0001 |
| 20 vs. 100 | -0.3142 | -0.3984 to -0.2300 | Yes | **** | < 0.0001 |
| 50 vs. 100 | -0.1031 | -0.1874 to -0.01891 | Yes | * | 0.0124 |
|  |  |  |  |  |  |
| AngII |  |  |  |  |  |
| 0 vs. 20 | -0.2047 | -0.2889 to -0.1204 | Yes | **** | < 0.0001 |
| 0 vs. 50 | -0.2489 | -0.3331 to -0.1647 | Yes | **** | < 0.0001 |
| 0 vs. 100 | -0.2590 | -0.3433 to -0.1748 | Yes | **** | < 0.0001 |
| 20 vs. 50 | -0.04422 | -0.1285 to 0.04001 | No | ns | 0.4830 |
| 20 vs. 100 | -0.05436 | -0.1386 to 0.02987 | No | ns | 0.3068 |
| 50 vs. 100 | -0.01014 | -0.09437 to 0.07409 | No | ns | 0.9870 |
|  |  |  |  |  |  |
| TF+Ang II |  |  |  |  |  |
| 0 vs. 20 | -0.1435 | -0.2277 to -0.05926 | Yes | *** | 0.0005 |
| 0 vs. 50 | -0.1099 | -0.1941 to -0.02566 | Yes | ** | 0.0073 |
| 0 vs. 100 | -0.1736 | -0.2578 to -0.08933 | Yes | **** | < 0.0001 |
| 20 vs. 50 | 0.03360 | -0.05063 to 0.1178 | No | ns | 0.6927 |
| 20 vs. 100 | -0.03007 | -0.1143 to 0.05416 | No | ns | 0.7594 |
| 50 vs. 100 | -0.06367 | -0.1479 to 0.02056 | No | ns | 0.1865 |

Ang II= angiotensin II, TF= exogenous transferrin

post hoc test of analysis of Rac-1 expression of HASMCs (two-way ANOVA)

| "Number of families" | 3 |  |  |  |  |
| --- | --- | --- | --- | --- | --- |
| "Number of comparisons per family" | 6 |  |  |  |  |
| Alpha | 0.05 |  |  |  |  |
|  |  |  |  |  |  |
| "Tukey's multiple comparisons test" | Mean Diff. | 95% CI of diff. | Significant? | Summary | Adjusted P Value |
|  |  |  |  |  |  |
| Blank |  |  |  |  |  |
| 0 vs. 20 | 0.1739 | 0.1149 to 0.2329 | Yes | **** | < 0.0001 |
| 0 vs. 50 | 0.2289 | 0.1700 to 0.2879 | Yes | **** | < 0.0001 |
| 0 vs. 100 | 0.2024 | 0.1434 to 0.2614 | Yes | **** | < 0.0001 |
| 20 vs. 50 | 0.05502 | -0.003956 to 0.1140 | No | ns | 0.0736 |
| 20 vs. 100 | 0.02849 | -0.03048 to 0.08746 | No | ns | 0.5520 |
| 50 vs. 100 | -0.02653 | -0.08550 to 0.03245 | No | ns | 0.6079 |
|  |  |  |  |  |  |
| AngII |  |  |  |  |  |
| 0 vs. 20 | 0.1098 | 0.05079 to 0.1687 | Yes | *** | 0.0002 |
| 0 vs. 50 | 0.1276 | 0.06865 to 0.1866 | Yes | **** | < 0.0001 |
| 0 vs. 100 | 0.1049 | 0.04591 to 0.1639 | Yes | *** | 0.0003 |
| 20 vs. 50 | 0.01786 | -0.04111 to 0.07683 | No | ns | 0.8371 |
| 20 vs. 100 | -0.004885 | -0.06386 to 0.05409 | No | ns | 0.9957 |
| 50 vs. 100 | -0.02275 | -0.08172 to 0.03623 | No | ns | 0.7141 |
|  |  |  |  |  |  |
| TF+Ang II |  |  |  |  |  |
| 0 vs. 20 | 0.03801 | -0.02096 to 0.09698 | No | ns | 0.3080 |
| 0 vs. 50 | 0.07614 | 0.01717 to 0.1351 | Yes | ** | 0.0080 |
| 0 vs. 100 | 0.06661 | 0.007639 to 0.1256 | Yes | * | 0.0227 |
| 20 vs. 50 | 0.03813 | -0.02084 to 0.09711 | No | ns | 0.3053 |
| 20 vs. 100 | 0.02860 | -0.03037 to 0.08758 | No | ns | 0.5488 |
| 50 vs. 100 | -0.009530 | -0.06850 to 0.04944 | No | ns | 0.9698 |

Ang II= angiotensin II, TF= exogenous transferrin

post hoc test of analysis of Cdc42 expression of HASMCs (two-way ANOVA)

| "Number of families" | 3 |  |  |  |  |
| --- | --- | --- | --- | --- | --- |
| "Number of comparisons per family" | 6 |  |  |  |  |
| Alpha | 0.05 |  |  |  |  |
|  |  |  |  |  |  |
| "Tukey's multiple comparisons test" | Mean Diff. | 95% CI of diff. | Significant? | Summary | Adjusted P Value |
|  |  |  |  |  |  |
| Blank |  |  |  |  |  |
| 0 vs. 20 | 0.01610 | -0.04289 to 0.07509 | No | ns | 0.8745 |
| 0 vs. 50 | -0.09769 | -0.1567 to -0.03871 | Yes | *** | 0.0007 |
| 0 vs. 100 | -0.1235 | -0.1825 to -0.06453 | Yes | **** | < 0.0001 |
| 20 vs. 50 | -0.1138 | -0.1728 to -0.05481 | Yes | *** | 0.0001 |
| 20 vs. 100 | -0.1396 | -0.1986 to -0.08063 | Yes | **** | < 0.0001 |
| 50 vs. 100 | -0.02582 | -0.08481 to 0.03317 | No | ns | 0.6281 |
|  |  |  |  |  |  |
| AngII |  |  |  |  |  |
| 0 vs. 20 | 0.09844 | 0.03945 to 0.1574 | Yes | *** | 0.0006 |
| 0 vs. 50 | 0.1202 | 0.06120 to 0.1792 | Yes | **** | < 0.0001 |
| 0 vs. 100 | 0.03678 | -0.02221 to 0.09577 | No | ns | 0.3357 |
| 20 vs. 50 | 0.02174 | -0.03725 to 0.08073 | No | ns | 0.7413 |
| 20 vs. 100 | -0.06167 | -0.1207 to -0.002676 | Yes | * | 0.0382 |
| 50 vs. 100 | -0.08341 | -0.1424 to -0.02442 | Yes | ** | 0.0035 |
|  |  |  |  |  |  |
| TF+Ang II |  |  |  |  |  |
| 0 vs. 20 | 0.08058 | 0.02159 to 0.1396 | Yes | ** | 0.0049 |
| 0 vs. 50 | 0.08756 | 0.02857 to 0.1466 | Yes | ** | 0.0022 |
| 0 vs. 100 | 0.1079 | 0.04890 to 0.1669 | Yes | *** | 0.0002 |
| 20 vs. 50 | 0.006984 | -0.05201 to 0.06597 | No | ns | 0.9877 |
| 20 vs. 100 | 0.02731 | -0.03168 to 0.08629 | No | ns | 0.5859 |
| 50 vs. 100 | 0.02032 | -0.03867 to 0.07931 | No | ns | 0.7783 |

Ang II= angiotensin II, TF= exogenous transferrin
