# Supplementary material for: Iron deficiency promotes aortic medial degeneration via destructing cytoskeleton of vascular smooth muscle cells
Source: Clin Transl Med. 2021 Jan 13;11(1):e276. doi: 10.1002/ctm2.276 (PMC7805404; doi:10.1002/ctm2.276)
Supplement: Supplementary file 4 — Supporting Information [file CTM2-11-e276-s004.doc]

| Survival Data summary | NC | Ang II | ID | ID +Ang II |
| --- | --- | --- | --- | --- |
| Number of rows | 48 | 48 | 48 | 48 |
| # of blank lines | 36 | 36 | 36 | 36 |
| # rows with impossible data | 0 | 0 | 0 | 0 |
| # censored subjects | 12 | 2 | 12 | 1 |
| # deaths/events | 0 | 10 | 0 | 11 |
|  |  |  |  |  |
| Median survival | Undefined | 20 | Undefined | 12.5 |

statistical analysis for survival curve

Ang II= angiotensin II, ID= iron deficiency

Comparison of survival curves between groups

| Survival Curve comparison |  |
| --- | --- |
| Comparison of Survival Curves |  |
|  |  |
| Log-rank (Mantel-Cox) test (recommended) |  |
| Chi square | 60.22 |
| df | 3 |
| P value | <0.0001 |
| P value summary | **** |
| Are the survival curves sig different? | Yes |
|  |  |
| Logrank test for trend (recommended) |  |
| Chi square | 15.77 |
| df | 1 |
| P value | <0.0001 |
| P value summary | **** |
| Sig. trend? | Yes |
|  |  |
| Gehan-Breslow-Wilcoxon test |  |
| Chi square | 0.03461 |
| df | 3 |
| P value | 0.9983 |
| P value summary | Ns |
| Are the survival curves sig different? | No |

Ang II= angiotensin II, ID= iron deficiency
