# Supplementary material for: Iron deficiency promotes aortic medial degeneration via destructing cytoskeleton of vascular smooth muscle cells
Source: Clin Transl Med. 2021 Jan 13;11(1):e276. doi: 10.1002/ctm2.276 (PMC7805404; doi:10.1002/ctm2.276)
Supplement: Supplementary file 5 — Supporting Information [file CTM2-11-e276-s005.doc]

Grade of aortic aneurysm or dissection

| Grade | AngII(n) | ID+AngII(n) | ID(n) | NC(n) |
| --- | --- | --- | --- | --- |
| Grade 1 | 3 | 1 | 6 | 6 |
| Grade 2 | 3 | 1 | 0 | 0 |
| Grade 3 | 0 | 2 | 0 | 0 |
| Grade 4 | 0 | 2 | 0 | 0 |

Grade 1:no aneurysm/dissection; Grade 2: an aneurysm/dissection; Grade 3: two aneurysms/dissections; Grade 4: dissection of the entire aorta
