# Supplementary figures and images for: Iron deficiency promotes aortic medial degeneration via destructing cytoskeleton of vascular smooth muscle cells
Source: Clin Transl Med. 2021 Jan 13;11(1):e276. doi: 10.1002/ctm2.276 (PMC7805404; doi:10.1002/ctm2.276)

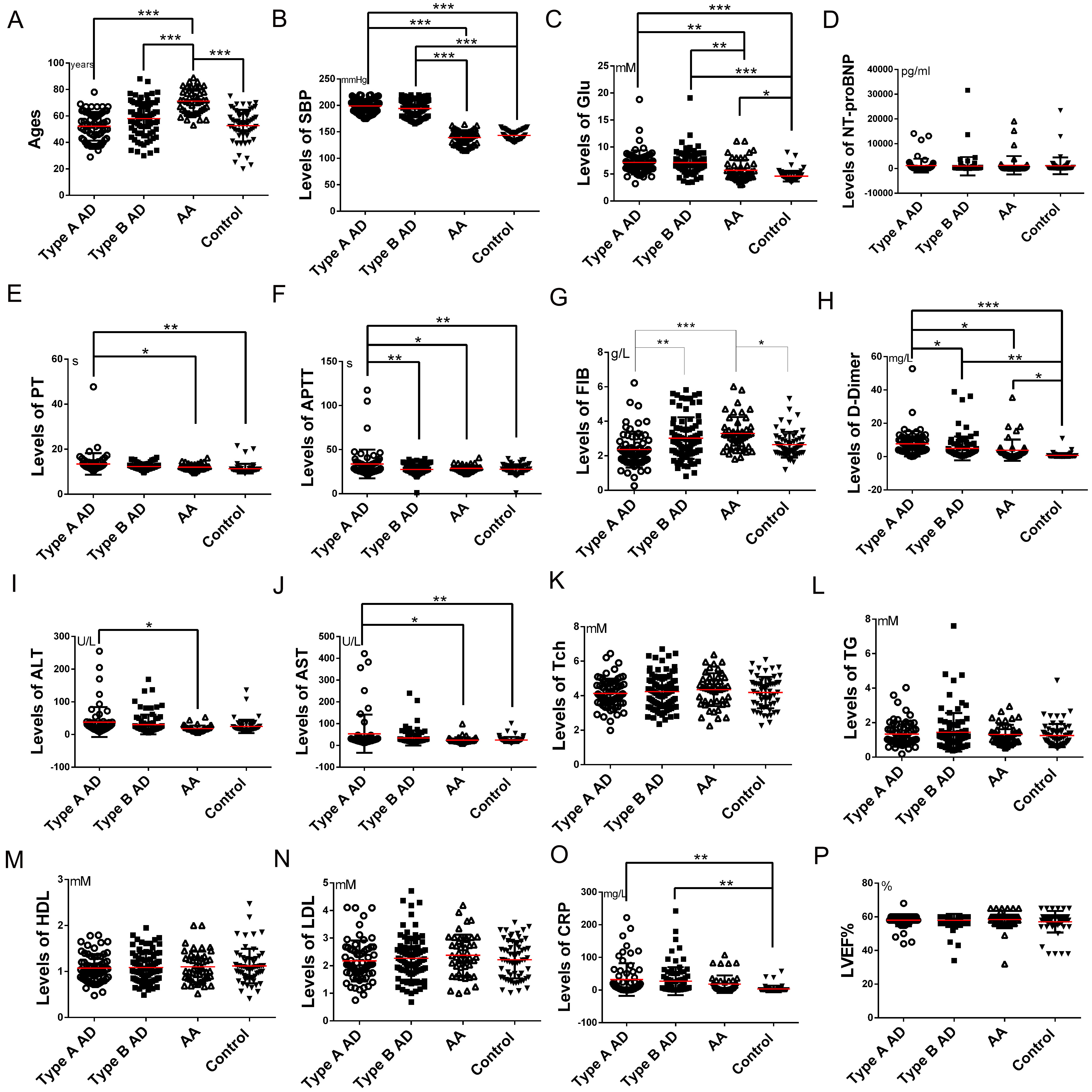

Supplement: Supplementary file 6 — Supporting Information [file CTM2-11-e276-s006.jpg]

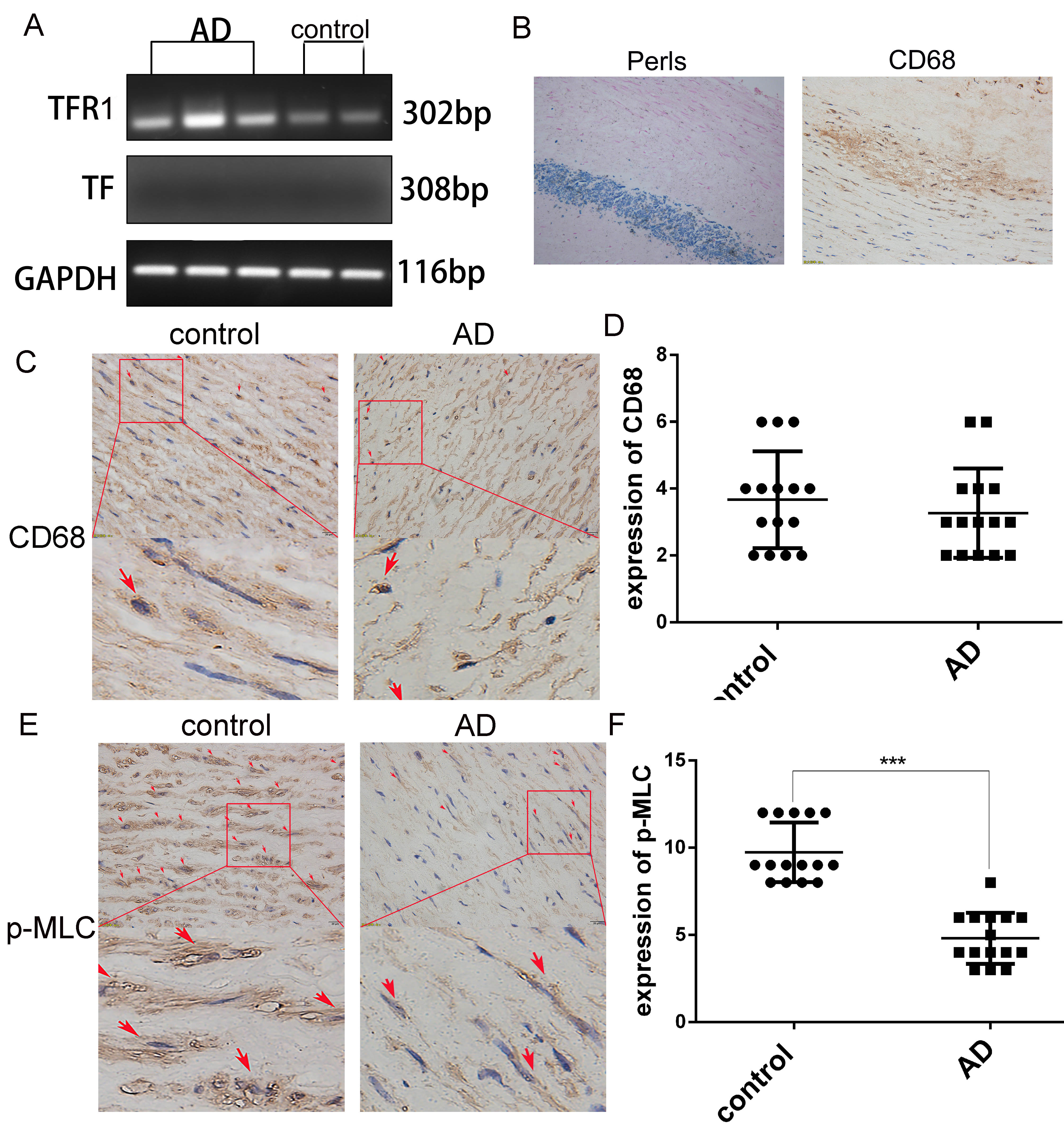

Supplement: Supplementary file 7 — Supporting Information [file CTM2-11-e276-s007.jpg]

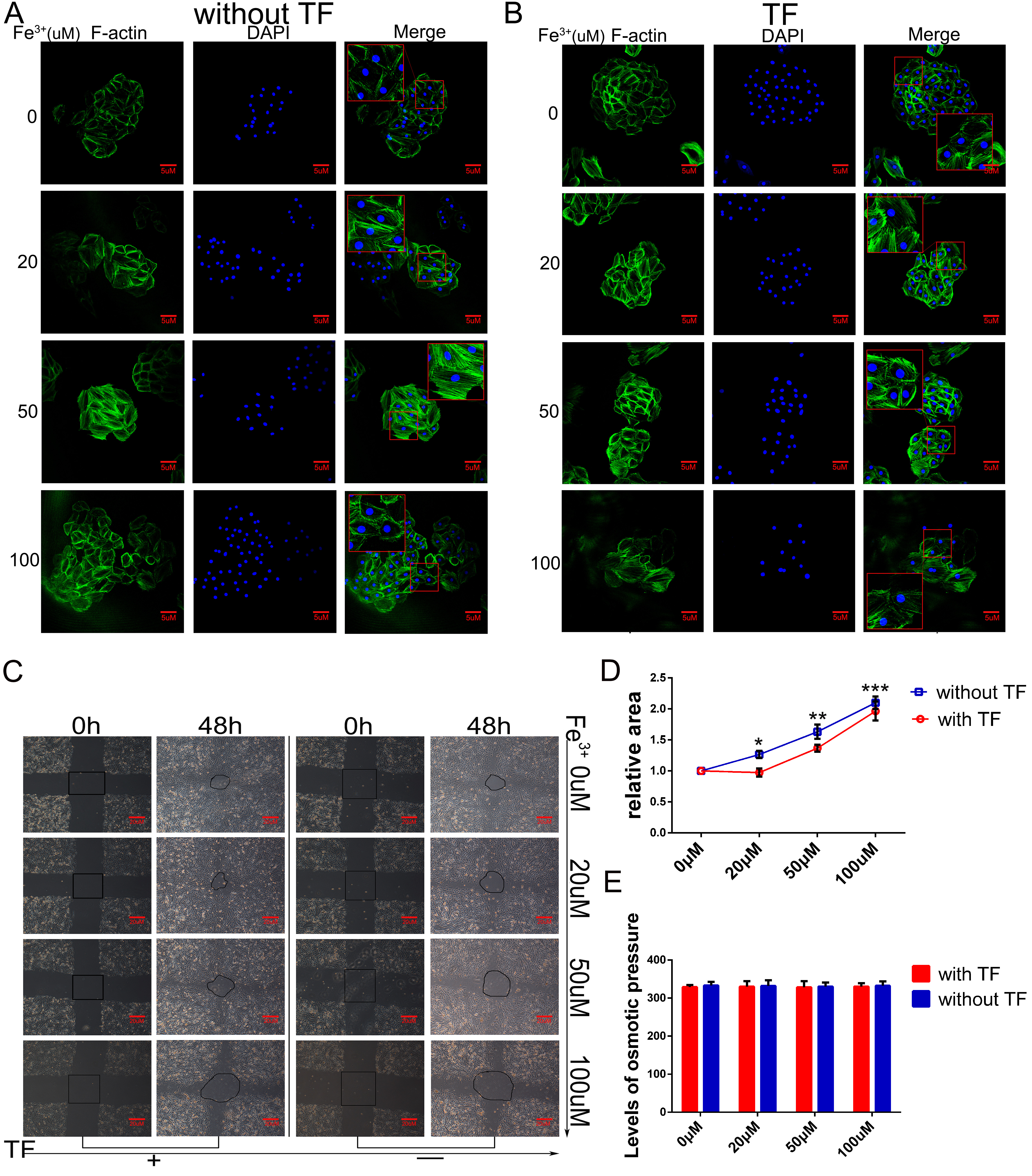

Supplement: Supplementary file 8 — Supporting Information [file CTM2-11-e276-s008.jpg]

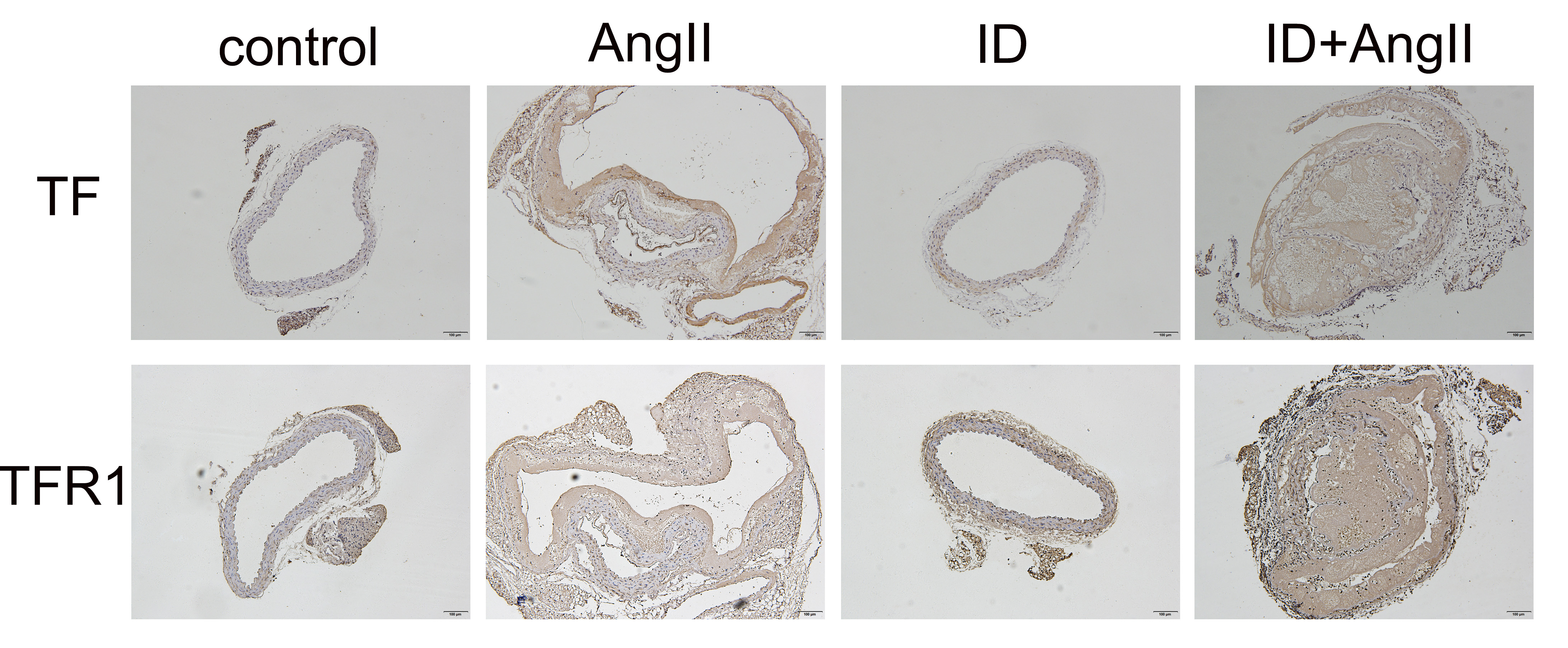

Supplement: Supplementary file 9 — Supporting Information [file CTM2-11-e276-s009.jpg]

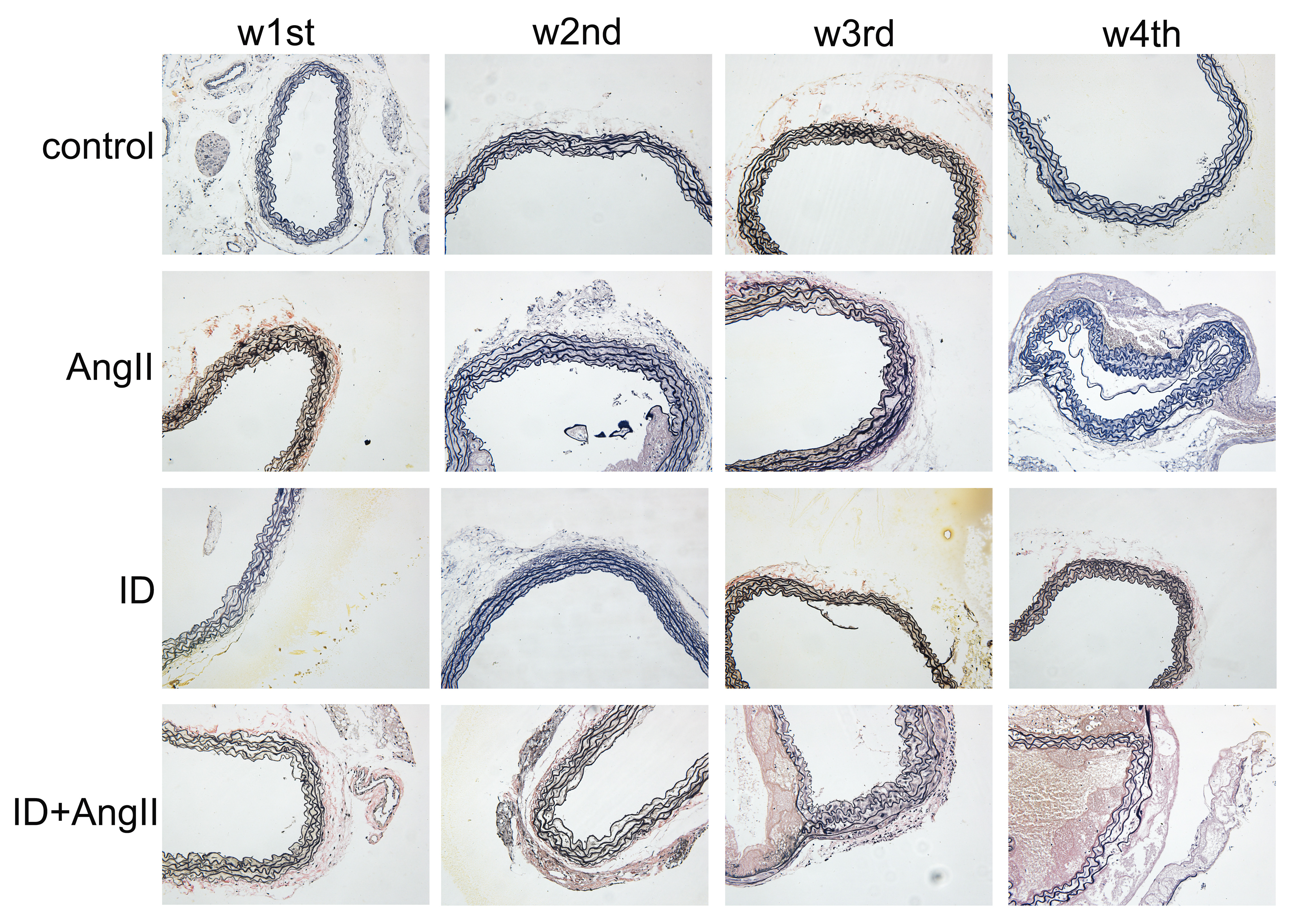

Supplement: Supplementary file 10 — Supporting Information [file CTM2-11-e276-s010.jpg]

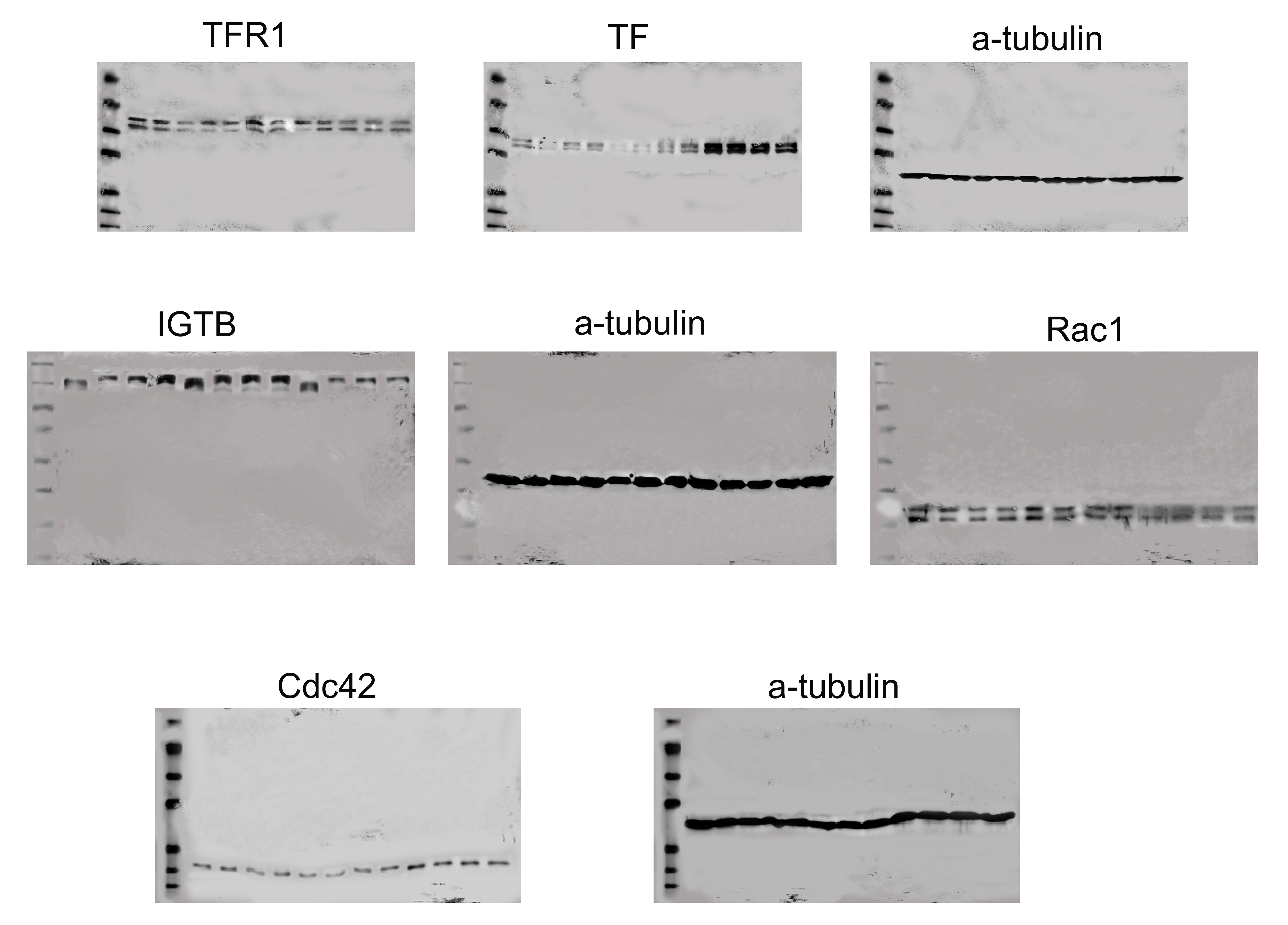

Supplement: Supplementary file 11 — Supporting Information [file CTM2-11-e276-s011.jpg]

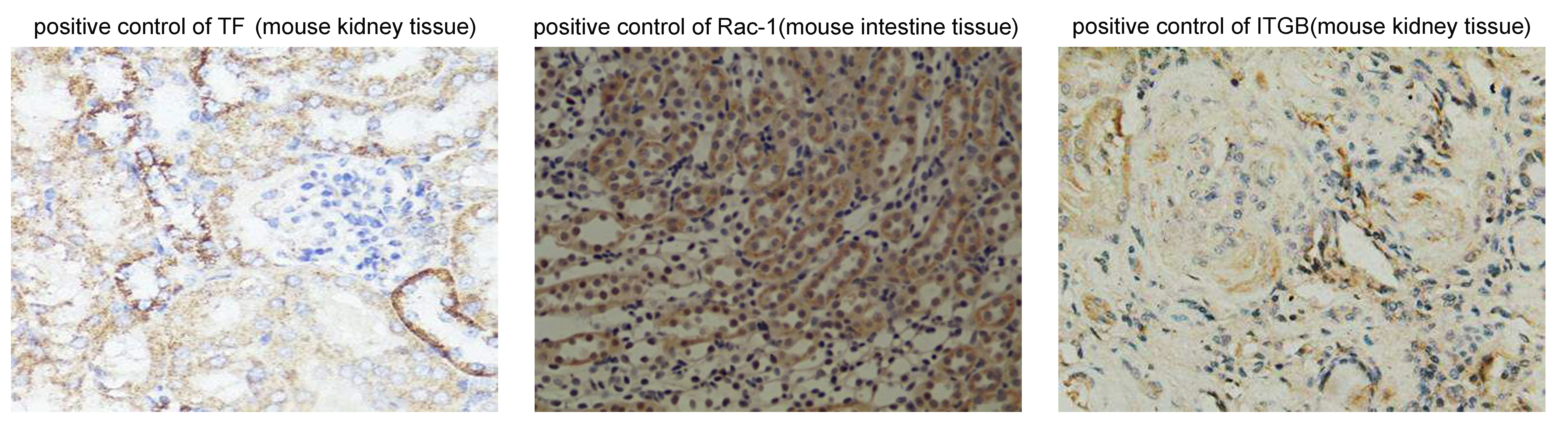

Supplement: Supplementary file 12 — Supporting Information [file CTM2-11-e276-s012.jpg]

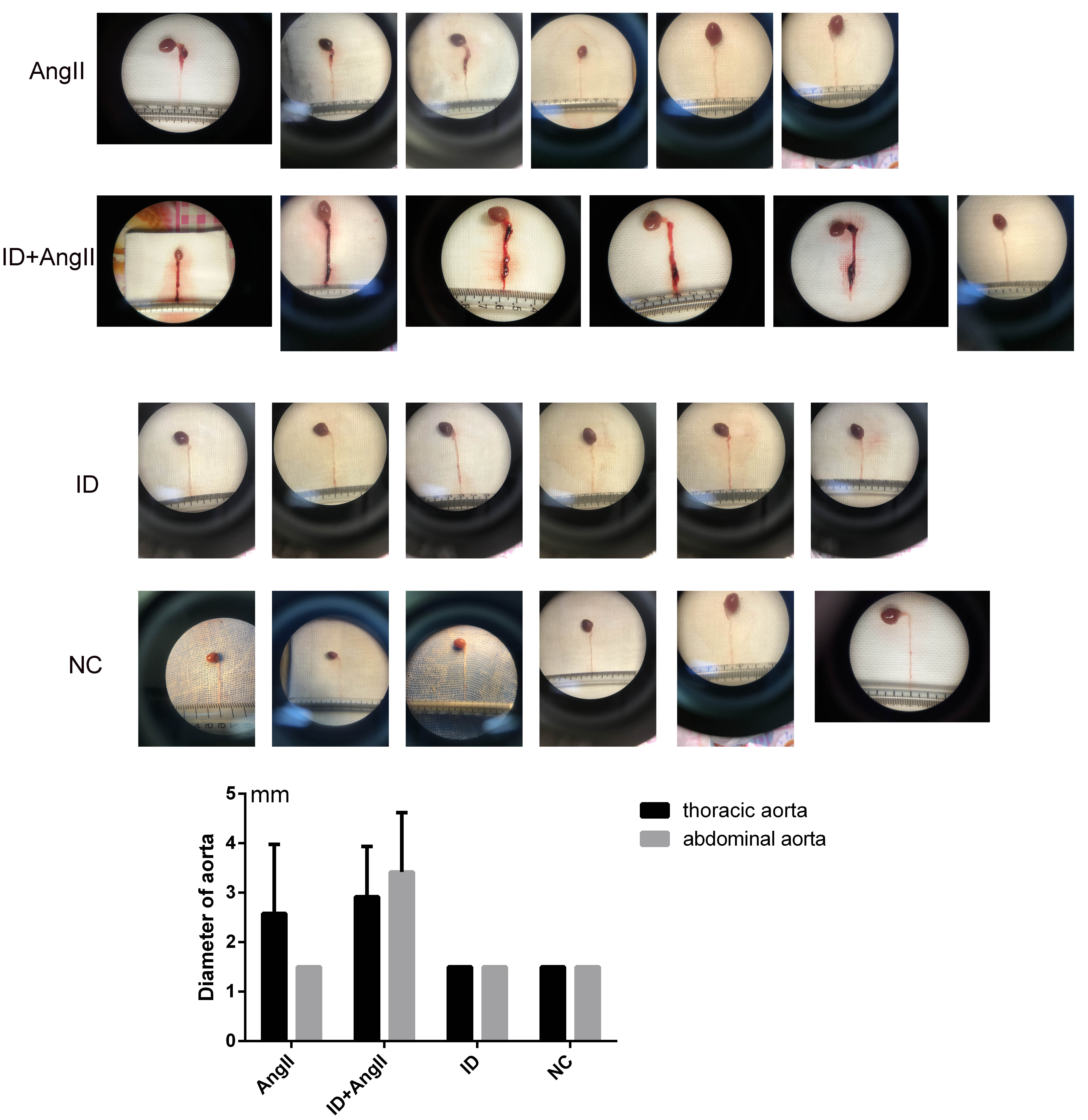

Supplement: Supplementary file 13 — Supporting Information [file CTM2-11-e276-s013.jpg]
